# Transcriptome-wide transmission disequilibrium analysis identifies novel risk genes for autism spectrum disorder

**DOI:** 10.1371/journal.pgen.1009309

**Published:** 2021-02-04

**Authors:** Kunling Huang, Yuchang Wu, Junha Shin, Ye Zheng, Alireza Fotuhi Siahpirani, Yupei Lin, Zheng Ni, Jiawen Chen, Jing You, Sunduz Keles, Daifeng Wang, Sushmita Roy, Qiongshi Lu

**Affiliations:** 1 Department of Statistics, University of Wisconsin-Madison, Madison, Wisconsin, United States of America; 2 Department of Biostatistics and Medical Informatics, University of Wisconsin-Madison, Madison, Wisconsin, United States of America; 3 Vaccine and Infectious Disease Division, Fred Hutchinson Cancer Research Center, Seattle, Washington, United States of America; 4 Department of Computer Sciences, University of Wisconsin-Madison, Madison, Wisconsin, United States of America; 5 University of Wisconsin-Madison, Madison, Wisconsin, United States of America; 6 Waisman Center, University of Wisconsin-Madison, Madison, Wisconsin, United States of America; 7 Center for Demography of Health and Aging, University of Wisconsin-Madison, Madison, Wisconsin, United States of America; Stanford University, UNITED STATES

## Abstract

Recent advances in consortium-scale genome-wide association studies (GWAS) have highlighted the involvement of common genetic variants in autism spectrum disorder (ASD), but our understanding of their etiologic roles, especially the interplay with rare variants, is incomplete. In this work, we introduce an analytical framework to quantify the transmission disequilibrium of genetically regulated gene expression from parents to offspring. We applied this framework to conduct a transcriptome-wide association study (TWAS) on 7,805 ASD proband-parent trios, and replicated our findings using 35,740 independent samples. We identified 31 associations at the transcriptome-wide significance level. In particular, we identified *POU3F2* (p = 2.1E-7), a transcription factor mainly expressed in developmental brain. Gene targets regulated by *POU3F2* showed a 2.7-fold enrichment for known ASD genes (p = 2.0E-5) and a 2.7-fold enrichment for loss-of-function *de novo* mutations in ASD probands (p = 7.1E-5). These results provide a novel connection between rare and common variants, whereby ASD genes affected by very rare mutations are regulated by an unlinked transcription factor affected by common genetic variations.

## Author summary

Autism spectrum disorder is a neurodevelopmental disorder with complex genetic etiology. Mutational variant studies link damaging and typically rare variants in protein-coding genes with disease outcomes, while genome-wide association studies identify genetic variations that are common in the human population associated with autism risk. Interestingly, studies targeting common and rare variants have implicated distinct risk pathways for autism. Here, we introduce a novel statistical framework for risk gene mapping, i.e., TITANS, to better analyze common genetic variants from parent-proband trios. TITANS integrates transmission disequilibrium information with tissue-specific regulatory annotations of multiple linked variants to infer risk genes. We pinpoint a novel autism gene *POU3F2*, which encodes a key transcription factor regulating multiple autism risk genes implicated in exome sequencing studies. Our findings provide a novel connection between rare and common variants, whereby autism genes affected by rare mutations are regulated by an unlinked transcription factor affected by common genetic variations.

## Introduction

Autism spectrum disorder (ASD [MIM: 209850]) is a highly heritable neurodevelopmental disorder affecting 1.5% of the world population [1]. It manifests as impaired social interaction and communication, repetitive behavior, and restricted interests with highly heterogenous clinical presentations [2]. Whole-exome sequencing (WES) studies for ASD have identified numerous ultra-rare or *de novo* single-nucleotide variants, small insertions and deletions (indels), and copy number variants (CNVs) [3–7]. Although these protein-disrupting genetic variations have large effects on the disease risk, they are only found in a moderate proportion of ASD probands. It has been estimated that the contribution of *de novo* loss-of-function mutations and CNVs to the variance in ASD liability was only 3% while common genetic variants explain 50% of the variance in the population [8]. Recently, genome-wide association studies (GWAS) with large sample sizes, coupled with novel statistical genetic approaches, have provided new insights into the involvement of common single-nucleotide polymorphisms (SNPs) in ASD. For instance, polygenic risk of ASD is significantly over-transmitted from parents to ASD probands but not their unaffected siblings in simplex families [9]. Such over-transmission was also observed in probands with *de novo* mutations in known ASD genes. Additionally, a recent GWAS meta-analysis of 18,381 ASD cases and 27,969 controls identified multiple genome-wide significant loci, but did not implicate apparent associations at ASD risk genes identified in WES studies [10]. These results suggested that distinct mechanistic pathways may underlie the ASD risk attributed to rare and common genetic variants, but our understanding of their interplay remains incomplete.

One potential approach to better dissect the genetic basis of ASD is to fine-map candidate genes affected by common SNPs and then investigate how they interact with genes harboring rare pathogenic variants implicated in WES studies. Transcriptome-wide association study (TWAS) is an analytical strategy that integrates expression quantitative trait loci (eQTL) annotations with GWAS data to identify disease genes [11–13]. Through advanced predictive modeling for gene expression traits, TWAS effectively combines association evidence across many eQTL in diverse tissues and has identified risk genes for numerous complex diseases [14].

**Fig 1:**
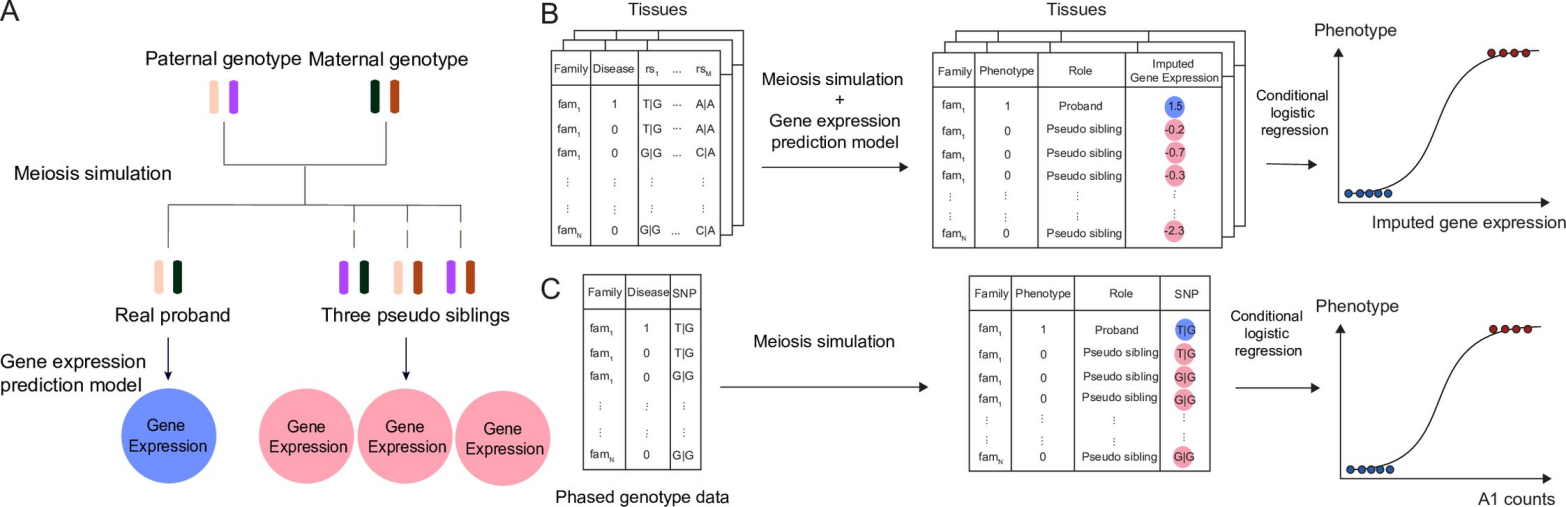
TITANS workflow. (A) We generate three matched pseudo siblings for each proband using the phased genotype data of parents and impute gene expression values. (B) We compare the impute gene expression traits between probands and matched pseudo siblings and use conditional logistic regression to quantify the associations. (C) We simulate genotype data for matched pseudo siblings and use conditional logistic regression to assess SNP-disease associations. A1 count stands for the counts of the minor allele.

In this study, we introduce TITANS (TrIo-based Transcriptome-wide AssociatioN Study) (**Material and Methods**), a novel statistical framework to conduct TWAS in proband-parent trios. TITANS uses a pseudo sibling matching procedure conceptually similar to classic trio-based GWAS approaches and is thus more robust to population stratification compared to population-based case-control studies [15]. Combining recent advances in TWAS methodology and the trio-based study design in multiple ASD cohorts, TITANS leverages multi-SNP transmission disequilibrium to robustly infer disease genes. Specifically, we performed a TWAS with eQTL and splicing quantitative trait loci (sQTL) in 12 brain tissues from the Genotype-Tissue Expression (GTEx) project [16] and the CommonMind consortium (CMC) [17]. We also took advantage from variant-based pseudo sibling matching [18–20], a protocol related to transmission equilibrium test (TDT) [21, 22] but with improved statistical power and robustness, and proposed a gene-based 3-pseudo-sibling design. For each proband, we generated 3 pseudo siblings using phased genotype data of the parents (**Fig 1A**). We imputed gene expression and intron usage values [23] for all probands and pseudo siblings (**Fig 1B**) using UTMOST [12] (10 GTEx brain tissues) and FUSION [11] (CMC dorsolateral prefrontal cortex; DLPFC) imputation models. We used conditional logistic regression [24] to assess the transmission disequilibrium of imputed gene expression traits while adjusting for the genetic similarity between proband and pseudo siblings. We also used the same framework to perform trio-based GWAS (**Fig 1C; Material and Methods**).

We demonstrate transmission disequilibrium of genetically regulated gene expression in brain tissues from parents to ASD probands. Specifically, we conducted GWAS and TWAS on 7,805 ASD trios from the Autism Genome Project (AGP), the Simons Simplex Collection (SSC), and the Simons Foundation Powering Autism Research for Knowledge (SPARK) cohort, and replicated our findings in an independent cohort of 13,076 cases and 22,664 controls (**Material and Methods, S1 and S2 Tables**). We identified 31 associations at the transcriptome-wide significance level. In particular, we identified *POU3F2* (MIM: 600494), a master regulator highly expressed in developmental brain whose downstream target genes are strongly enriched for known ASD genes and mutations.

## Results

### Transmission disequilibrium of polygenic risk, gene expression, and SNP alleles

We applied multiple analytical approaches to dissect common SNPs’ contributions to ASD risk at different scales. First, we performed pTDT [9] to examine the transmission disequilibrium of ASD polygenic risk in probands. ASD polygenic risk scores (PRS) were constructed using case-control samples from the iPSYCH cohort (N = 35,740; **Material and Methods**). We confirmed a highly significant over-transmission of ASD PRS from parents to probands in multiple datasets (p = 1.4E-25 in the meta-analysis), including the SPARK cohort which has not been previously analyzed (p = 1.0E-11; **S1 Fig**). No significant over-transmission was identified in 3,245 healthy siblings (p = 0.88).

**Fig 2:**
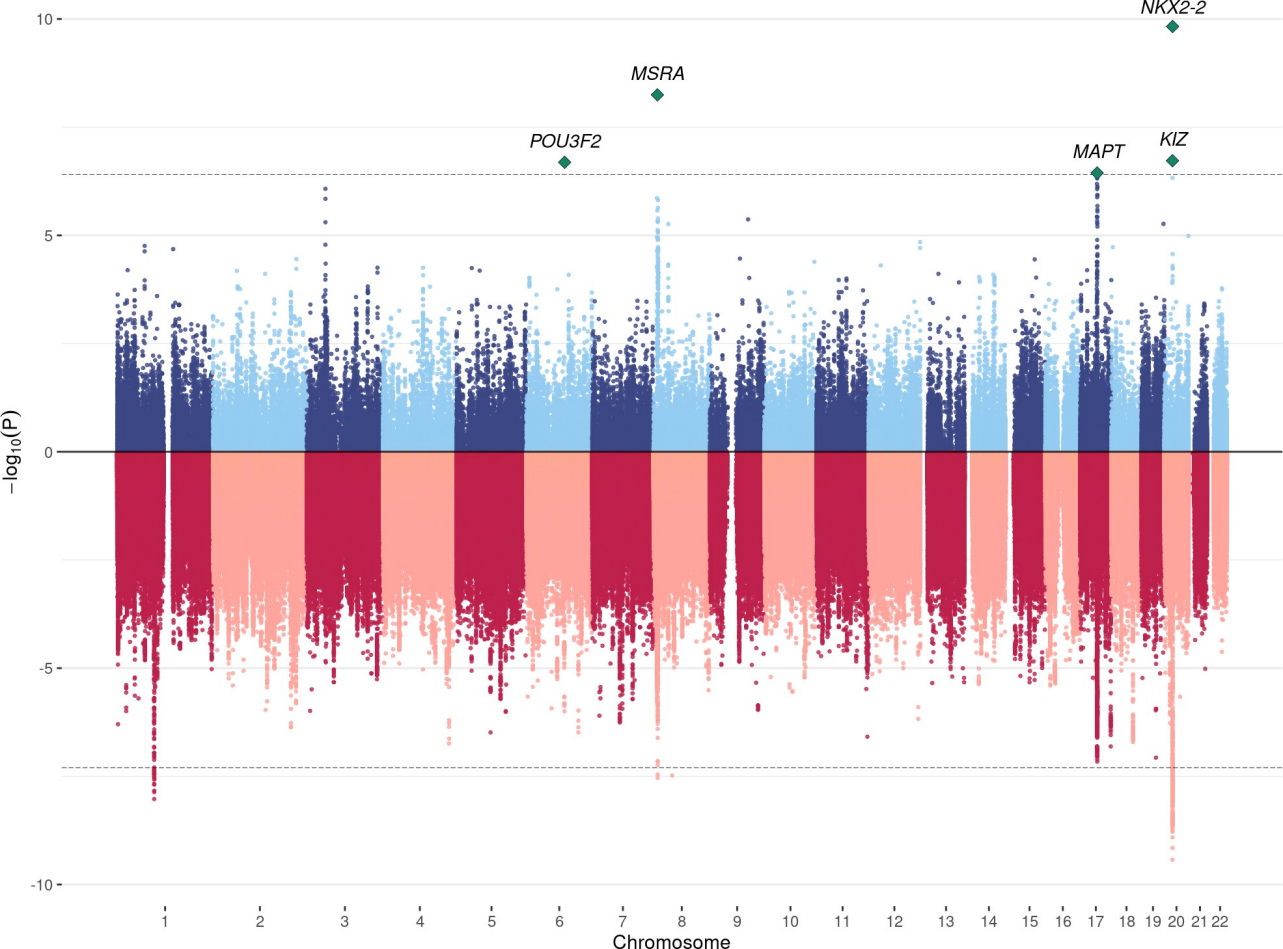
Mirrored Manhattan plot for TWAS and GWAS results. TWAS results are shown in the upper panel. GWAS associations are shown in the lower panel. The dashed line in the upper panel indicates the cross-tissue transcriptome-wide significance cutoff (p = 4.0E-7) and the dashed line in the lower panel is the genome-wide significance cutoff (p = 5.0E-8). TWAS associations for all 12 tissues are shown.

We identified significant transmission disequilibrium of *POU3F2* expression (p = 5.6E-7, cross-tissue adjusted p = 0.035; GTEx hippocampus) and *MSRA* (MIM: 601250) intron usage (p = 2.3E-7, cross-tissue adjusted p = 0.028; CMC DLPFC splicing) in 7,805 trios after correcting for the number of genes in each tissue (**Tables 1** and S1). Both associations were replicated in an independent cohort of 13,076 cases and 22,664 controls (p = 0.015 and 0.002, respectively). Meta-analysis enhanced the associations at *POU3F2* and *MSRA* and identified 29 additional significant associations at the transcriptome-wide significance level (**S1 Table** and **S2–S11 Figs)**. Five associations, i.e. *POU3F2* (p = 2.1E-7), *MSRA* (p = 5.7E-9), *MAPT* (MIM: 157140) (p = 3.6E-7), *KIZ* (MIM: 615757) (p = 1.9E-7), and *NKX2-2* (MIM: 604612) (p = 1.5E-10), remained significant after a stringent Bonferroni correction for all genes and all tissues in the analysis (**Table 1** and **Fig 2**). In total, these associations implicated 18 unique candidate genes from 7 loci, including 5 novel loci not previously identified in GWAS.

**Table 1:** Cross-tissue significant associations in TWAS. Beta and SE indicate the normalized effect size estimates and standard error in conditional logistic regression. Some effect size estimates are unavailable in the replication cohort since FUSION does not provide effect size estimates.

			Discovery Stage (N = 7,805 trios)			Replication Stage (N = 35,740)			Meta-analysis		
Gene	Chr	Tissue	Beta	SE	P	Beta	SE	P	Beta	SE	P
*POU3F2*	6	GTEx hippocampus	0.09	0.02	5.56E-07	0.03	0.01	0.015	0.05	0.01	2.05E-07
*MSRA*	8	CMC DLPFC—splicing	0.09	0.02	2.26E-07	-	-	0.002	-	-	5.67E-09
*MAPT*	17	CMC DLPFC—splicing	0.06	0.02	2.42E-04	-	-	4.09E-04	-	-	3.62E-07
*KIZ*	20	CMC DLPFC	0.05	0.02	1.73E-03	-	-	2.62E-05	-	-	1.88E-07
*NKX2-2*	20	GTEx nucleus accumbens basal ganglia	-0.05	0.02	2.44E-03	-0.07	-0.01	2.91E-09	-0.06	0.01	1.49E-10

We performed extensive analyses to demonstrate the robustness and well-controlled type-I error of TITANS and validate the association results. We first examined if genotype imputation error or inaccurate gene expression imputation could inflate type-I error. Hard genotype calls and dosages produced highly consistent gene expression imputation results (**S12 Fig**). We also added random noises to imputed gene expressions and showed that inaccurate gene expression imputation does not inflate type-I error rate in TITANS (**S13** and **S14 Figs**). Next, no significant associations were identified in unaffected sibling-parent trios (**S15 Fig)** or after randomly shuffling probands and pseudo siblings (**S16 Fig**), suggesting well-controlled type-I error in TITANS. Finally, we compared TITANS with two alternative trio-based approaches which contrast probands with parental data and one-sibling control generated from non-transmitted parental alleles, respectively (**Material and Methods**). TITANS showed superior statistical power in both simulations and analyses of real data (**S17–S19 Figs**).

GWAS meta-analysis of trios and case-control cohorts identified 4 genome-wide significant loci (**S2 Table**), 3 of which (1p21.3, 8p23.1, and 20p11.23) were among previously identified loci [10]. A locus on chromosome 8 is novel but we note that the top SNP did not exist in the trio-based analysis. Overall, TWAS identified significant genes at multiple known ASD loci but also pinpointed novel ASD loci without significant signal in GWAS (**Fig 2**). Two GWAS loci on chromosomes 8 and 20 were also identified in TWAS. No significant associations were found in sibling-parent trios (**S15 Fig**).

### Candidate risk genes and gene set enrichment analysis

**Fig 3:**
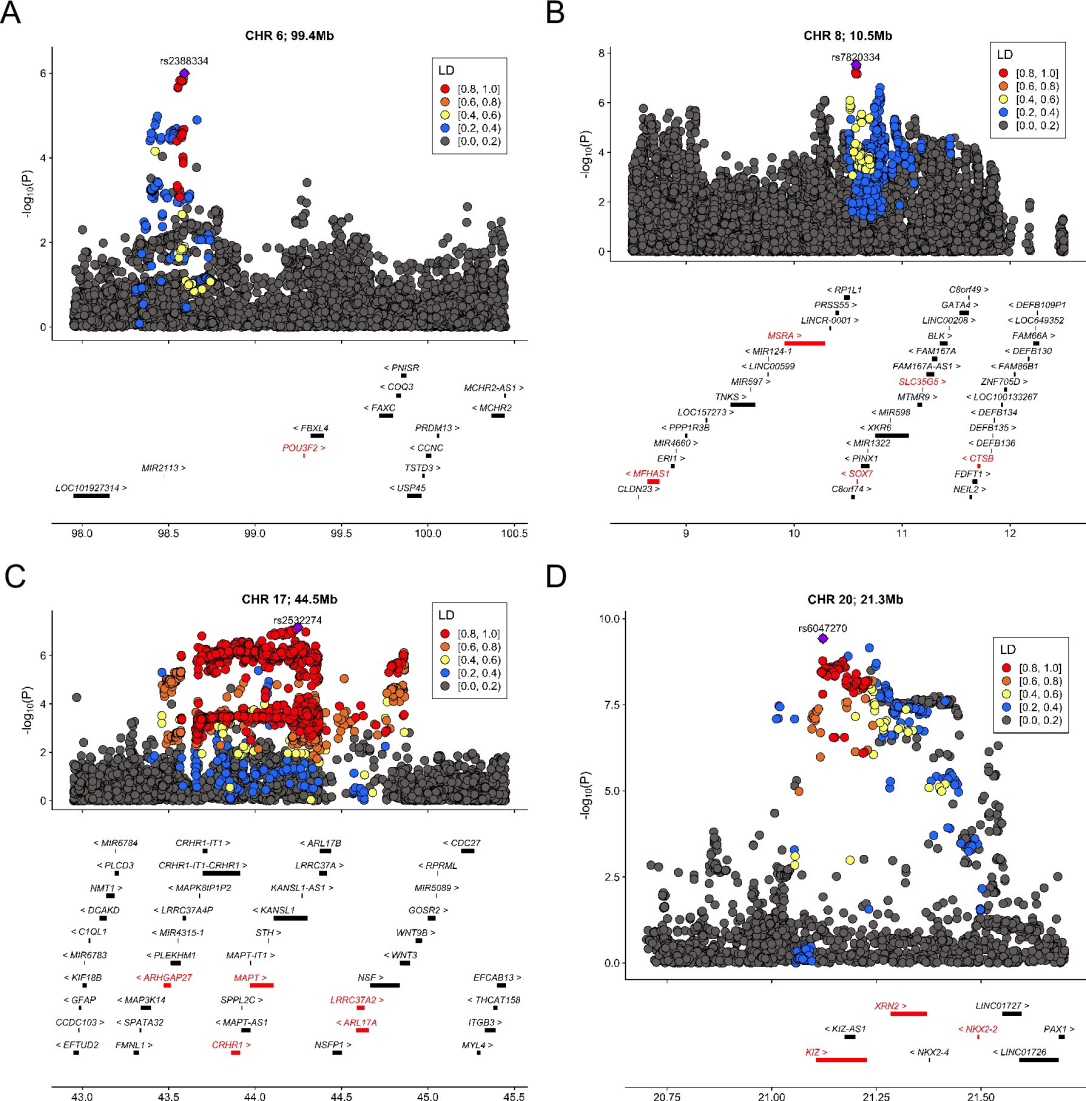
Significant loci identified in TWAS. We identified 5 cross-tissue transcriptome-wide significant associations from 4 loci. (A) Chromosome 1, 99.4 mb (B) Chromosome 8, 10.5 mb (C) Chromosome 17, 44.5 mb (D) Chromosome 20, 21.3 mb. For each locus, the index SNP with the most significant association in GWAS is marked as purple diamond and the color of data points indicates linkage disequilibrium (LD) of neighboring SNPs with the index SNP. Genes are highlighted in red if they reached transcriptome-wide significance in at least one tissue. The x-axis denotes genome coordinates and the y-axis denotes association p-values in GWAS.

Among the 5 significant genes after a stringent Bonferroni correction for all genes and all tissues in the analysis (**Figs 3** and **S20**), *POU3F2* (also known as *BRN2*) is primarily expressed in the central nervous system (**S21 Fig**), especially in hippocampus and hypothalamus [25]. It encodes a transcription factor with important roles in neurogenesis and brain development [26, 27]. It is a known risk gene for bipolar disorder [28, 29] and has been identified as a master regulator of gene expression changes in schizophrenia and bipolar disorder [27, 30]. Deletions resulting in loss of one copy of *POU3F2* cause a disorder of variable developmental delay, intellectual disability, and susceptibility to obesity [31]. Heterozygous *POU3F2* knockout mice showed deficits in adult social behavior [32] and it has been linked to neural proliferation phenotypes in stem cell models of ASD [33]. Although this locus did not reach genome-wide significance in the GWAS, gene-level association at *POU3F2* was supported by a SNP-level association peak 700 kb upstream of *POU3F2* (**Fig 3A**; lead SNP rs2388334, p = 1.0E-6).

Other association findings also have support from the literature for their involvement in psychiatric disorders. *MAPT* encodes the microtubule-associated protein tau known to associate with multiple neurodegenerative diseases including Alzheimer’s disease (MIM: 104300) and Parkinson’s disease (MIM: 605909) [34] and balance of *MAPT* isoforms is critical for neuronal normal functioning [35]. This locus showed suggestive associations in the GWAS (lead SNP rs2532274, p = 6.9E-8). *KIZ*, *NKX2-2*, and *MSRA* are located at 2 loci previously identified in ASD GWAS [10]. *KIZ* encodes the Kizuna centrosomal protein which is critical for stabilizing mature centrosomes during spindle formation [36]. *NKX2-2* encodes the homeobox protein NKX2.2, a transcription factor with an essential role in interpreting graded Sonic hedgehog signals and selecting neuronal identity [37]. *MSRA* shows high levels of expression in the human central nervous system and *Msra* knockout mice show abnormal behaviors [38, 39].

We performed conditional analysis using 7,805 ASD trios (**Material and Methods**). Our analysis suggests that *DDHD2* (P = 2.68E-5) and *CTSB* (P = 0.002) may independently contribute to ASD risk in DLPFC and the alternative splicing of *MSRA* (chr8:10163257:10177393:clu_45644, P = 0.002) may be the driver association on chromosome 8 (**S3 Table**). Our analysis did not reveal a clear candidate at the *MAPT* locus on chromosome 17, 44.5 mb, possibly due to multicollinearity caused by extensive LD at this locus.

**Fig 4:**
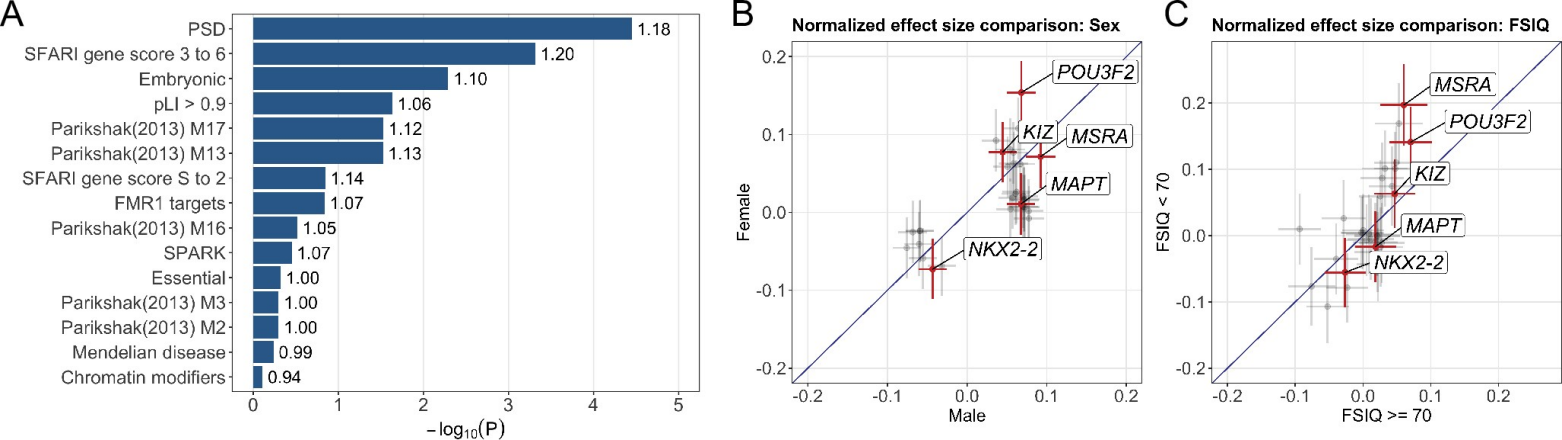
Gene set enrichment analysis and subgroup TWAS results. (A) Enrichment -log10 p-values for different gene sets are shown in the bar plot. Fold enrichment values are labeled next to each bar. (B) The normalized effect size estimates in sex-stratified TWAS. Effects of 31 associations identified in the pooled TWAS are shown in the plot. Five cross-tissue significant associations are highlighted in red. For each cross, the interval indicates normalized effect ± standard error. A diagonal suggestive line passing through the origin is also included. (C) The normalized effect size estimates in FSIQ-stratified TWAS. Each interval indicates normalized effect ± standard error. A diagonal suggestive line passing through the origin is also included.

Next, we compared TWAS findings and ASD risk genes identified in rare variant studies. We investigated if genes with nominal associations (p < 0.05) in TWAS are enriched in known ASD pathways. Among the 15 gene sets we tested (**Material and Methods**), only genes encoding postsynaptic density proteins (PSD; enrichment = 1.18, p = 3.6E-5) and SFARI genes with evidence score 3–6 (enrichment = 1.20, p = 4.8E-4) showed significant enrichment for TWAS findings after multiple testing correction (**Fig 4A** and **S4 Table**). Additionally, we note that some genes with weaker evidence in the SFARI Gene database [40] were identified using samples from the AGP and SSC cohorts and thus may not represent independent evidence. Notably, gene sets that are known to harbor significant burden of rare or *de novo* variants in ASD, e.g. *FMR1* target genes (enrichment = 1.07, p = 0.14), SFARI genes with evidence score S-2 (enrichment = 1.13, p = 0.14), and chromatin modifier genes (enrichment = 0.94, p = 0.77), showed negligible enrichment for TWAS associations. These results confirmed the distinct etiologic pathways underlying common and rare genetic variations in ASD.

### TWAS associations in subgroups

Further, we investigated if the effects of candidate genes are consistent in different phenotypic subgroups. We applied TITANS to assess the 31 associations identified in TWAS in sample subgroups stratified by sex and full-scale intelligence quotient (FSIQ) [7, 9]. In sex-stratified analysis of 6,484 male probands and 1,321 female probands, most genes showed comparable effect sizes in males and females (correlation = 0.65; **Fig 4B**). Cross-tissue significant genes *POU3F2*, *KIZ*, and *NKX2-2* had higher effects in females. Of note, *POU3F2* showed a 2.26-fold ratio (p = 0.026, permutation test) between its effects in females and in males, reaching statistical significance even under a substantially smaller sample size of female probands (**S5 Table**). This is consistent with a female protection mechanism that requires a larger effect size and risk load. We next performed FSIQ-stratified analysis and compared the transmission disequilibrium in probands with higher (FSIQ > = 70, N = 2,127) and lower FSIQ (FSIQ < 70, N = 731). The effect size estimates in two subgroups were mostly consistent (correlation = 0.71; **Fig 4C**). *POU3F2* showed a stronger effect in the subgroup with lower FSIQ (p = 0.023 in subgroup with higher FSIQ, p = 0.009 in subgroup with lower FSIQ), with a 2-fold effect difference (p = 0.036, permutation test).

### Regulatory role of *POU3F2* in ASD

**Fig 5:**
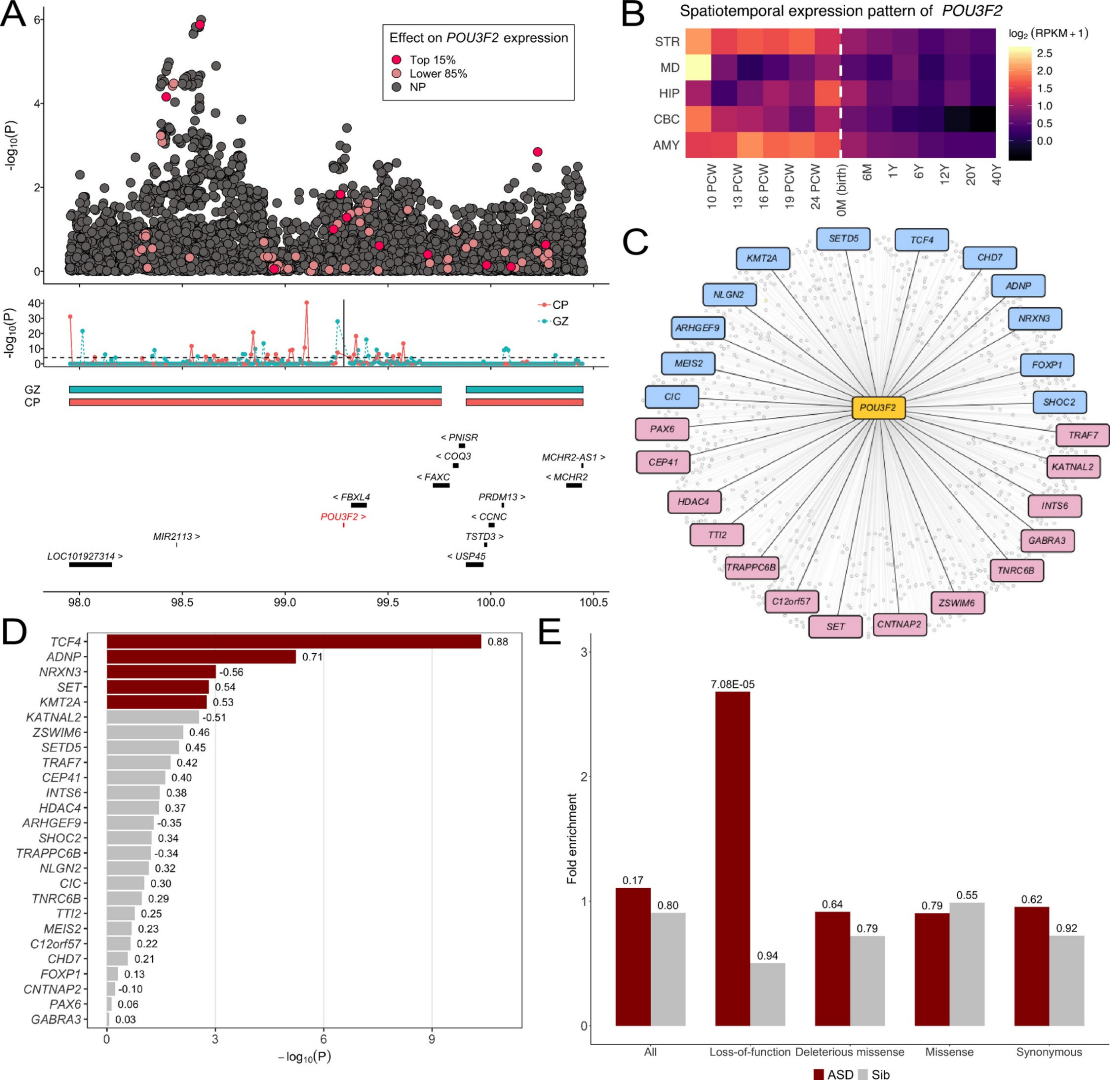
Biological underpinnings of *POU3F2*. (A) The upper panel shows GWAS associations at the *POU3F2* locus. Predictor SNPs in the *POU3F2* imputation model highlighted in red or pink based on their effect size rankings (top 15% or lower 85%). The middle panel shows the TADs in CP and GZ zones and the Hi-C interactions between each 10-kb bin in the region and *POU3F2* promoter which is indicated by the vertical line. The lower panel lists the genes at this locus. (B) The spatiotemporal expression pattern of *POU3F2* in 12 developmental stages across 5 brain regions. The periods span fetal development, infancy, childhood, adolescence, and adulthood, from 4 post-conceptional weeks (PCW) to 40 postnatal years (Y). Average log_2_ of reads per kilo base per million mapped reads (RPKM)+1 value for samples of the same region and developmental stage are shown. The dashed line indicates the boundary between later fetal and early infancy stages (0 month). (C) Transcription factor target genes of *POU3F2*. ASD genes in the SPARK gene list are highlighted in blue and additional genes with SFARI evidence score S to 2 are highlighted in pink. (D) Coexpression between ASD genes and *POU3F2* in hippocampus. The -log10 p-values for testing coexpressions are shown in the plot. The correlation coefficients between ASD genes and *POU3F2* are labeled next to each bar. Genes reaching the Bonferroni-corrected statistical significance are colored in red. (E) Enrichment of *de novo* mutations in 1,013 *POU3F2* targets. Enrichment results in 2,508 ASD probands and 1,911 unaffected siblings across four annotation categories (all mutations, loss-of-function, missense, deleterious missense, and synonymous) are shown. p-values are shown above each bar.

The transcription factor encoded by *POU3F2* is a key regulator in multiple psychiatric disorders [27, 30]. Based on its robust association with ASD in our analysis and the absence of protein-altering mutations in ASD probands, we hypothesized that *POU3F2* may also play a central role in ASD through its regulatory network. We investigated the biological underpinnings of *POU3F2* by leveraging diverse types of genomic data. First, we confirmed the link between the gene-level association at *POU3F2* and GWAS associations in the same region through integrating fetal brain Hi-C data from the germinal zone (GZ) and postmitotic-zone cortical plate (CP) [41]. *POU3F2* and the GWAS association peak 700 kb upstream are located in the same topological associating domain (TAD) that is conserved in both GZ and CP zones (chr6: 97.52–99.76 mb; **Fig 5A**). Additionally, we identified 59 non-overlapping bins, each of 10 kb in size and within 1 mb from the transcription start site of *POU3F2*, showing significant interactions with the promoter region of *POU3F2* (p < 1.0E-4; **Material and Methods**; **S6–S8 Tables**). Multiple bins showing significant interactions with *POU3F2* promoter colocalized with GWAS associations in this region. For example, SNP rs62422661 (p = 2.0E-5 in GWAS) is located in the bin located at 98.54–98.55 mb on chromosome 6 which significantly interacts with *POU3F2* in the CP zone (p = 2.0E-12). In addition, 15 SNP predictors for *POU3F2* expression, including 2 strong predictors with effect sizes ranked at top 15%, are located in bins interacting with *POU3F2* promoter (**Fig 5A**).

Next, we examined the spatiotemporal expression pattern of *POU3F2* in 5 brain regions, i.e. cerebellar cortex (CBC), striatum (STR), hippocampus (HIP), mediodorsal nucleus of thalamus (MD), and amygdala (AMY), spanning from fetal development to adulthood [42] (**Material and Methods**). *POU3F2* showed significantly elevated expression in developmental brains compared to postnatal brains across all 5 brain regions (p = 5.3E-3, permutation test; **Fig 5B**). A similar pattern was also observed in several other genes (e.g. *MAPT*) while *NKX2-2* showed elevated expression in postnatal brains (**S22 Fig**).

Additionally, we used the regulatory network from Chasman *et al*. [43] to investigate the enrichment of known ASD genes in target genes regulated by *POU3F2*. The transcription factor target network of *POU3F2* contained 1,013 genes (**Fig 5C** and **S9 Table**) in neuro progenitor cells. Among 1,013 *POU3F2* targets, 26 genes overlapped with SPARK genes (i.e. 153 curated genes known to be associated with autism) or SFARI genes with scores S to 2 [40] (**Material and Methods)**. These genes showed strong enrichment (enrichment = 2.1, p = 0.012) for the SPARK genes and for SFARI genes with scores S to 2 (enrichment = 2.66, p = 2.0E-5). Furthermore, 5 out of 26 regulated ASD genes showed significant coexpression with *POU3F2* in hippocampus after multiple testing correction (Pearson’s correlation coefficient test; **Fig 5D** and **S10 Table**), significantly more than what is expected by chance alone (p = 4.0E-4, permutation test). Many remaining ASD genes also showed moderate evidence of coexpression with *POU3F2*.

Various gene sets previously shown to enrich for rare and *de novo* mutations in ASD, including chromatin modifiers (p = 2.6E-4), *FMR1* targets (p = 0.009), and loss-of-function intolerant genes (p = 2.2E-6), were significant enriched in *POU3F2* targets (**S9 Table**). Furthermore, *POU3F2* target genes were significantly enriched for loss-of-function *de novo* mutations (enrichment = 2.68, p = 7.1E-5, Poisson test; **Material and Methods**) in 2,508 SSC probands (**Fig 5D** and **S11 Table**). Enrichment remained substantial with suggestive statistical evidence even after we removed known ASD genes in either the SPARK gene list or SFARI genes with scores S to 2 from the analysis (enrichment = 1.75, p = 0.04) (**S23 Fig and S12 Table**). Further, we observed substantially weaker enrichment for loss-of-function mutations in target genes of 950 other transcription factors (p = 0.015, one-sided Poisson test), suggesting that such enrichment is specific to *POU3F2*.

Finally, we obtained TFBS of *POU3F2* based on the prior network in Chasman *et al*. [43], and used LDSC to assess the enrichment of ASD heritability in these TFBS [44] (**Material and Methods**). SNPs located near *POU3F2* binding sites explained 11.7% of ASD heritability, showing a 5.3-fold enrichment with moderate statistical evidence (p = 0.054; **S13 Table**).

## Discussion

In this study, we have presented TITANS, an analytical framework for testing the transmission disequilibrium of genetically regulated molecular traits between parents and probands. Through integrative modeling of GWAS data in trios and rich QTL annotations from large consortia such as GTEx [16], this approach effectively combines association evidence at multiple SNPs to implicate novel risk genes affected by common genetic variations. It extends the classic SNP-level TDT analysis to quantify the transmission disequilibrium of genetically imputed gene expression from parents to probands. Compared to existing TWAS approaches, our method leverages the trio-based design to enhance the robustness and interpretability of association findings.

Our approach enjoys well-calibrated type-I error, suggested by extensive simulations and real-data analysis. Applied to multiple large-scale ASD cohorts including the SPARK study which has not been previously reported, we conducted a TWAS on 7,805 proband-parent trios and replicated our findings in 35,740 case-control samples. Meta-analysis identified a total of 31 transcriptome-wide significant associations, with 5 novel loci not previously implicated in GWAS.

Among the identified associations, convergent evidence suggested a critical etiologic role of *POU3F2* in ASD. *POU3F2* encodes a transcription factor which mainly expresses in the central nervous system [26] and has known key regulatory roles in schizophrenia and bipolar disorder [27, 30]. In our analysis, it reached transcriptome-wide statistical significance in trio-based TWAS and was successfully replicated in the case-control replication. Furthermore, meta-analysis strengthened the association at *POU3F2*, and it remained significant after a stringent multiple testing correction for all genes and all tissues analyzed in this study. Subtype analysis suggested that *POU3F2* has enhanced over-transmission in female probands (2.3-fold) and individuals with lower FSIQ (2-fold). Furthermore, we demonstrated its etiologic importance and its connection to other ASD risk genes through integrative analysis of diverse types of genomic data. Analysis of fetal brain Hi-C data confirmed significant interactions between *POU3F2* promoter and multiple genome regions near GWAS associations located in the same TAD. Analysis of spatiotemporal gene expression data suggested significantly elevated *POU3F2* expression in developmental brain. TFBS of *POU3F2* were enriched for ASD heritability. Downstream target genes regulated by *POU3F2* were enriched for known ASD risk genes identified in WES studies. *POU3F2* targets were also significantly enriched for loss-of-function *de novo* mutations in ASD probands. Enrichment remained substantial even after known ASD genes were removed from the gene set. To our knowledge, this is the first time *POU3F2* is implicated as an ASD risk gene, showcasing TITANS’ ability to identify novel risk genes that cannot be implicated by traditional case-control GWAS.

We note that TITANS inherited TWAS’ limitations [14]. Statistical power in TWAS is determined by many factors including technical issues such as the quality of gene expression imputation [14]. In our study, we have used the UTMOST method [12], a state-of-the-art approach that utilizes cross-tissue eQTL information to improve expression imputation in rarer tissue types. Still, accurate imputation remains challenging given the moderate sample size of brain transcriptomic data from GTEx and CommonMind. Although TITANS suggested the role that hippocampus played on ASD (**Table 1** and **Fig 5D**), we do not rule out the involvement of other brain regions and developmental stages. In our analysis. *POU3F2* achieved the highest imputation quality (R^2^ = 0.21) in hippocampus compared to other brain regions [12], which is consistent with the higher disease association of *POU3F2* in hippocampus (**S14 Table**). Although a strong association in TWAS may hint at a mechanistic role of the identified gene in the given tissue, a lack of association does not suggest that the tissue and disease is truly unassociated. Pinpointing the tissue- and temporal-specific role of ASD risk genes in both pre- and post-natal brains is an important future direction. Also, many associations in our meta-analysis only reached transcriptome-wide significance, instead of experiment-wide significance. The lack of power in our analysis was not only due to low imputation accuracy but lack of ASD samples. We need future replication to confirm the role of those associations. Finally, although trio-based analysis is robust to population stratification, our analysis focused on individuals with European descents only due to the poor trans-ethnic portability of gene expression imputation performance [45]. It remains unclear how these associations will replicate in other populations.

WES studies have identified numerous extremely rare, protein-disrupting variants in ASD and have implicated risk genes and pathways [3–7]. Successful studies focusing on other types of genetic variants using GWAS and whole-genome sequencing approaches have just begun to emerge [9, 10, 46–48]. A common and somewhat puzzling observation in these studies was that common SNPs associated with ASD did not influence the same genes and pathways enriched for rare variants. Our analysis partly confirmed this observation–genes showing strong associations in TWAS had limited overlap with genes identified through WES. However, the *POU3F2* results provide a clear example of the direct link of genes affected by very rare mutations with common genetic variations at a second, unlinked locus. These findings provide insights into the interplay of common and rare genetic variations in ASD, shed light on regulatory network-based modeling of epistatic interactions, and have broad implications for the genetic basis of other diseases.

## Material and methods

### Sample information and data processing

We accessed AGP samples through dbGaP (accession: phs000267). The total sample size was 7,880. Genotyping was performed using the Illumina Human 1M-single Infinium BeadChip. Details on these samples have been described elsewhere [49, 50]. We accessed samples from the SSC and the SPARK study through the Simons Foundation Autism Research Initiative (SFARI) [51, 52]. The SSC cohort contains comprehensive genotype and phenotype information from 2,600 simplex families, each family has one ASD child, and healthy parents and siblings. Genotyping was performed in batches by the Illumina IMv1, IMv3 Duo, and Omni2.5 arrays. Details on these data can be found on the SFARI website and have been described elsewhere [48, 51]. Samples in the SPARK study were genotyped by the Illumina Infinium Global Screening Array. Details on these samples have been previously reported [53, 54] and are available on the SFARI website [52].

We performed pre-imputation quality control (QC) using PLINK [55]. Only individuals with self-reported European ancestries were included in the study. SNPs with genotype call rate < 0.95, minor allele frequency (MAF) less than 0.01, or significant deviation from Hardy-Weinberg equilibrium (p < 1.0E-6) were removed from the analysis. Samples with genotype missing rate > 0.05 were also excluded from the analysis. We used genetic relationship coefficients estimated from GCTA [56] to identify and remove overlapped samples among different cohorts. After QC, 2,188, 1,794, and 3,823 independent proband-parent trios remained in AGP, SSC, and SPARK cohorts respectively. 1,432 and 1,813 trios of sibling-parent trios remained in SSC and SPARK. The UCSC liftOver tool was used to liftover the genome coordinates in AGP samples from hg18 to hg19. The genotype data were phased and imputed to the HRC reference panel version r1.1 2016 using the Michigan Imputation server [57]. We removed SNPs with imputation quality < 0.8 or MAF < 0.01 in the post-imputation QC. 7,260,224 SNPs remained in the AGP study after QC. 7,298,961 SNPs, 7,029,817 SNPs, and 6,866,248 SNPs remained in the SSC 1Mv1, 1Mv3, and Omni2.5 datasets, respectively. 7,031,717 SNPs remained in the SPARK data.

We used case-control samples from the iPSYCH cohort as the replication dataset in our study (13,076 cases and 22,664 controls). The iPSYCH ASD sample contains all Danish children born between 1981 and 2005 and details on this cohort are described elsewhere [58]. This cohort has been included in a recent ASD GWAS meta-analysis [10]. Samples in the iPSYCH cohort are independent from samples in the AGP, SSC, and SPARK.

### Polygenic transmission disequilibrium analysis

We used the iPSYCH GWAS summary statistics as the training dataset to generate ASD polygenic risk score (PRS) on samples from the AGP, SSC, and SPARK cohorts. We performed a LD-clumping using PLINK with a p-value threshold of 1, a LD threshold of 0.1, and a distance threshold of 1,000 kb. After clumping, 167,085 SNPs remained in the dataset. PRSice was used for PRS calculation [59]. We quantified the transmission disequilibrium of ASD PRS using the pTDT approach [9].

### Trio-based TWAS and GWAS analysis

We developed a statistical framework TITANS to perform trio-based TWAS (**Fig 1B**). We used UTMOST [12] gene expression imputation models for 10 brain tissues in GTEx and imputation models for CMC DLPFC expression and intron usage values implemented in FUSION [11]. UTMOST model uses a cross-tissue penalized regression model to borrow information from tissues with larger sample size and improve imputation accuracy of gene expression [12]. FUSION trains multiple imputation models in each tissue separately, including Bayesian sparse linear mixed model, elastic net, LASSO, and an ordinary least square model using single best eQTL as the predictor. We selected the best model using the cross-validation.

Given a gene with *m* predictor SNPs, we extracted those SNPs from parents’ phased genotypes and recombined the chromosomes based on Mendelian inheritance to create the genotypes of pseudo siblings (**Fig 1A**). Since only *cis*-regulators within the local region are included in gene expression and intron usage imputation models, we assumed no crossover events in our analysis. Given the parental data, four recombined pseudo offspring genotypes can be created, each having a paternal haplotype and a maternal haplotype. We imputed gene expression and intron usage on each proband and all four simulated pseudo siblings. We excluded the pseudo sibling whose imputed expression is the closest to the proband’s since one of the four simulated offsprings’ genotype should be identical to the proband if there is no phasing error or crossover. We tested the association between imputed gene expression and disease phenotype using conditional logistic regression [24] (**Fig 1B**), with conditional likelihood

$$L=\Pi_{i=1}^{N}\frac{\exp\left(x_{pi}\beta \right)}{\exp\left(x_{pi}\beta \right)+\exp\left(x_{s1i}\beta \right)+\exp\left(x_{s2i}\beta \right)+\exp\left(x_{s3i}\beta \right)}$$

Here, *x*_*pi*_,*x*_*s*1*i*_,…,*x*_*s*3*i*_ denote the imputed gene expression or intron usage values of the proband and 3 pseudo siblings in the i^th^ family, with *N* families in total. We used the clogit function in the R package ‘survival’ to numerically estimate the effect size β, which can be interpreted as transmission disequilibrium of imputed expression. The SE of β, the z-score test statistic, and association p-value are also reported. TWAS was conducted in the AGP, SSC, and SPARK cohorts separately. Adjusted p-values were calculated using the Benjamini-Hochberg procedure to control the false discovery date (FDR) [60]. Results in different trio-based cohorts were meta-analyzed using the inverse-variance weighted method [61]. These results were then meta-analyzed with the associations in the replication stage using z-score-based meta-analysis weighted by sample sizes [61].

We performed TWAS in sample subgroups based on sex and FSIQ. We conducted sex-stratified TWAS in each cohort and meta-analyzed the result across AGP, SSC, and SPARK using the inverse-variance weighted method [61]. FSIQ-stratified analysis based on a cutoff of 70 was conducted in SSC and SPARK separately and then combined through meta-analysis. P-values for fold enrichment were obtained by permutation test. In each permutation, we randomly shuffled sex and FSIQ subgroup assignment in AGP, SSC, and SPARK cohorts and re-estimated enrichment. The fold enrichment estimate in real data was compared with the empirical null distribution based on 10,000 permutations to compute the p-values.

We used a similar framework to conduct GWAS in trios (**Fig 1C**). For each SNP, we create four recombined genotypes based on parental data, exclude a genotype identical to the proband’s genotype, and perform conditional logistic regression to assess the association between each SNP and ASD status.

### Alternative TWAS approaches using one-sibling controls and parental controls

We compared the three-sibling approach implemented in TITANS with two alternative approaches. The first alternative approach generates one pseudo sibling within each family using only non-transmitted parental alleles. The gene expression of pseudo sibling is the sum of parental gene expressions minus the proband’s gene expression. We used glm in base R to perform logistic regression and estimate the effect size β, the SE of β, the z-score test statistic, and association p-value. The second alternative approach performs conditional logistic regression on probands and parent controls. We used the clogit function in the R package ‘survival’ and reported the effect size β, the SE of β, the z-score test statistic, and association p-value for each gene.

We performed the alternative TWAS approaches on 7,805 trios in AGP, SSC, and SPARK in GTEx hippocampus and their shuffled data. For 1-sibling matching, we randomly assigned one sample from four members generated by 3-sibling matching (i.e. a proband and 3 pseudo siblings) as cases in each family, and constructed their pseudo siblings using untransmitted parental alleles. For parent-control matching, we selected one sample from the four members in 3-sibling matching in each family, and matched the them with their parents.

### Assessing the robustness of TITANS

We added random noises ε ~ *N*(0,*σ*^2^) to the genetically imputed gene expressions of *POU3F2* in hippocampus in 3,823 proband-parent trios from the SPARK cohort. We chose a grid of values for *σ*^2^ (i.e., *σ*^2^ ranging evenly from 0.05 to 1, with each grid size 0.05) to represent small to large technical noise. We then applied conditional logistic regression to test the disease association of *POU3F2* using these gene expressions with uncertainty. We repeated the whole procedure 100 times for each noise level *σ*^2^ and calculated the statistical power by averaging the counts of significant p-values (p < 0.05).

Next, we repeated the analysis at the transcriptome-wide scale. Similar to the first analysis, we added random noise ε ~ *N*(0,*σ*^2^) to the hippocampal expression of all genes using trios in the SPARK cohort. For each noise level *σ*^2^ (i.e. 0, 0.005, 0.1, 0.015, and 0.2), we performed TWAS to identify disease-associated genes.

Further, we randomly shuffled the phenotype status of 7,805 ASD probands and 23,415 matched pseudo-siblings in our analysis of AGP, SSC, and SPARK cohorts on 12 brain tissues. We applied TITANS to 3,245 trios of unaffected siblings and their parents as well.

Finally, we conducted simulations to compare the power of 3-sibling-matching, 1-sibling-matching, and parent-control approaches. We randomly sampled gene expression values for 1,000 parents-offspring trios from *N*(0, 1) and used a logistic model

$$P(D=1)=\frac{1}{1+\exp\left(-\beta_{0}-\beta_{1}\times G\right)}$$

to determine the disease status for offspring. Here, *D* and *G* denote the disease status and gene expressions, respectively, while *β*_0_ and *β*_1_ denote prevalence and effect parameters, respectively. Notably, the baseline disease prevalence is

$$\frac{1}{1+\exp\left(-\beta_{0}\right)}.$$

We considered the offspring to be affected by the disease when the modeled disease probability is greater than 0.5. We compared the power between disease under *β*_0_ equals 2.25 and -2.5. That is, the disease prevalence of 0.9 and 0.07, respectively, while the corresponding sample sizes are 900 trios and 7 trios.

### Conditional analysis

Since several loci harbor multiple candidate ASD genes, we performed conditional analysis using 7,805 ASD trios by incorporating multiple genes identified at the same loci in the same tissue in conditional logistic regression. We fine-mapped the associations on chromosome 8, 10.5 mb (CMC DLPFC and CMC DLPFC splicing) and on chromosome 17, 44.5 mb (CMC DLPFC, CMC DLPFC splicing, GTEX cerebellum, and GTEx nucleus accumbens basal ganglia) (**S3 Table**). In each family, we removed the pseudo sibling whose normalized imputed expression for the genes to be fine-mapped has the lowest sum of squared difference to the proband’s since one of the four simulated siblings should be identical to the proband if there is no phasing error or crossover. We performed inverse variance weighted method to meta-analyze results in different cohort.

### Gene set enrichment analysis

We used hypergeometric test to assess if genes with nominal TWAS associations (p < 0.05 in any tissue) were enriched in gene sets that have been linked to ASD in past literatures (**S3 Table**). Gene sets assessed in our analysis included co-expression modules M2, M3, M13, M16, and M17 from Parikshak *et al*. [62], *FMR1* (MIM: 309550) targets, genes encoding postsynaptic density proteins (PSD), gene preferentially expressed in human embryonic brains downloaded from BRAINSPAN [63], essential genes [64], chromatin modifier genes [5], and genes with probability of loss-of-function intolerance (pLI) > 0.9 from the Exome Aggregation Consortium [65]. In addition, we downloaded genes from the SFARI Gene database in August 2019 [40] and created two gene sets based on evidence scores. The gene set based on scores S, 1, or 2 include genes involved in ASD with high to suggestive evidence and genes predisposing to ASD in the context of a syndromic disorder. Genes with scores 3–6 have limited evidence or have only been hypothesized to link to ASD. Finally, we obtained a list of 153 genes with known roles in ASD curated by the SPARK study [66]. We refer to this gene set of SPARK genes in our analyses.

### Hi-C analysis

We used the human fetal brain Hi-C data (GEO: GSE77565) [41, 67] at resolution 10 kb in the analysis. The samples were sequenced using Illumina HiSeq 2000 chip, collecting from three individuals aging gestation week (GW) 17–18 (one sample from GW17 and two samples from GW18). The Hi-C libraries were constructed in two brain zones GZ and CP. The TAD region of GZ and CP are also provided. We converted the Hi-C contact matrices (HDF5 format) normalized by ICE [68] into the sparse contact matrix format (BED format) and leveraged Fit-Hi-C [69] to detect the significant interactions in the regions of interest. Benjamini-Hochberg procedure [60] was employed to control the false discovery rate.

### Spatiotemporal expression analysis

We obtained spatiotemporal gene expression data from BRAINSPAN for 17 candidate genes [63] with significant associations in our TWAS analysis. Average log_2_(RPKM+1) values for samples of the same region and developmental stage were calculated. Expression data were derived from 5 brain regions, i.e. CBC, STR, HIP, MD, and AMY, and spanned from 8 weeks post-conception (PCW) to 40 years as indicated in Kang *et al*. [70]. mRNA sequencing was performed using the Illumina Genome Analyzer IIx. Details on these data are described elsewhere [42].

### *POU3F2* transcription factor binding network

The transcriptional targets of *POU3F2* were obtained using the procedure from Chasman *et al*. [43]. We downloaded *POU3F2* motif position weight matrices (PWM) from 3 databases, CIS-BP [71], ENCODE [72], and JASPAR [73]. We obtained DNase-I seq data for neural progenitor cells from the Roadmap Epigenome Consortium [74] (GEO: GSE18927). Next, we applied the Protein Interaction Quantification (PIQ) algorithm [75] to identify *POU3F2* motif binding sites across the human genome. Using the DNase-I seq data, the PIQ algorithm defines a purity score (0.5–1.0) for a motif instance, which quantifies the likelihood of a true binding event in that site. PIQ motif instances were mapped to the transcription start sites from Gencode v10 within a 10 kb radius. The confidence of the edge between a transcription factor and the target was defined as the maximum PIQ purity score among all transcription factor motif instances and the target gene. Furthermore, the confidence score was converted to percentile ranks ranging from 0 to 1. Only edges with confidence score > 0.99 were preserved in the final network, containing 1,013 outgoing edges of *POU3F2*. We also obtained target genes for other 950 transcription factors using a similar procedure.

### Coexpression between ASD genes and *POU3F2* in hippocampus

We first defined ASD genes as genes in either SPARK genes or SFARI genes with scores S, 1, or 2, and there were 26 ASD genes regulated by *POU3F2* in neuro progenitor cells. We obtained the hippocampal expression of *POU3F2* and 26 ASD genes regulated by *POU3F2* from BRAINSPAN [63]. log_2_(RPKM+1) values for samples of the same region were calculated. We used the function rcorr in R package ‘Hmisc’ [76] to calculate the correlation coefficients between expression of ASD genes and *POU3F2*. We shuffled the sample IDs in gene expressions and obtained the p-value for coexpressing by calculating the proportion of permutations with a higher or equal number significantly coexpressed genes.

### *De novo* mutation enrichment analysis

We used published *de novo* mutability [77] of synonymous, missense, and loss-of-function variants to estimate the expected counts of mutations. Published *de novo* mutation data [5] in 2,508 probands and 1,911 controls from the SSC cohort were accessed through denovo-db [78]. Loss-of-function mutations were defined as frameshift, stop-gained, splice-donor, stop-gained near splice, frameshift near splice, stop-lost, or splice-acceptor mutations. Missense mutations included missense and missense-near-splice labels from the denovo-db. Synonymous mutations included synonymous and synonymous-near-splice labels. Variants with Missense badness, PolyPhen-2, and Constraint (MPC) [79] score greater than 2 are considered deleterious missense. We used ANNOVAR [80] to obtain MPC scores and we generated the deleterious missense mutability table using the mutational model in Samocha *et al*. [77]. Finally, we used Poisson test to assess enrichment and quantify the statistical evidence [77].

### Partitioned heritability analysis

We used stratified linkage disequilibrium score regression [44] (LDSC) to assess the partitioned ASD heritability in *POU3F2* transcription factor binding sites (TFBS). We used the PIQ motif instances we generated in the network analysis and expanded each TFBS by 100, 150, and 250 base pairs up- and downstream. Further, we partitioned the heritability from the using the meta-analyzed GWAS summary statistics as input. The model also included 53 LDSC baseline annotations, as recommended in Finucane *et al*. [44].

## Supporting information

S1 Fig Transmission disequilibrium of PRS in different cohorts. Transmission disequilibrium was quantified by the pTDT approach. Results in probands and unaffected siblings are highlighted in different colors. The mean pTDT deviation and the SE are shown. P-values are labeled above each interval.
(PNG)

S2 Fig Forest plot for the significant association in GTEx anterior cingulate cortex BA24. *LRRC37A2* reached transcriptome-wide significance in the TWAS in GTEx anterior cingulate cortex BA24. Standardized effect sizes (beta) and SEs are provided for all cohorts. Beta and SE in the discovery cohort are meta-analyzed results based on AGP, SSC, and SPARK. Beta and SE in the combined cohort are calculated from the meta-analysis of discovery and replication stages.
(PDF)

S3 Fig Forest plot for significant associations in GTEx caudate basal ganglia. *FBXW12* and *LRRC37A2* reached transcriptome-wide significance in the TWAS in GTEx caudate basal ganglia. Standardized effect sizes (beta) and SEs are provided for all cohorts. Beta and SE in the discovery cohort are meta-analyzed results based on AGP, SSC, and SPARK. Beta and SE in the combined cohort are calculated from the meta-analysis of discovery and replication stages.
(PDF)

S4 Fig Forest plot for significant associations in GTEx cerebellar hemisphere. *NME6* and *LRRC37A2* reached transcriptome-wide significance in the TWAS in GTEx cerebellar hemisphere. Standardized effect sizes (beta) and SEs are provided for all cohorts. Beta and SE in the discovery cohort are meta-analyzed results based on AGP, SSC, and SPARK. Beta and SE in the combined cohort are calculated from the meta-analysis of discovery and replication stages.
(PDF)

S5 Fig Forest plot for significant associations in GTEx cerebellum. *MAPT* and *LRRC37A2* reached transcriptome-wide significance in the TWAS in GTEx cerebellum. Standardized effect sizes (beta) and SEs are provided for all cohorts. Beta and SE in the discovery cohort are meta-analyzed results based on AGP, SSC, and SPARK. Beta and SE in the combined cohort are calculated from the meta-analysis of discovery and replication stages.
(PDF)

S6 Fig Forest plot for the significant association in GTEx hippocampus. *POU3F2* reached transcriptome-wide significance in the TWAS in GTEx hippocampus. Standardized effect sizes (beta) and SEs are provided for all cohorts. Beta and SE in the discovery cohort are meta-analyzed results based on AGP, SSC, and SPARK. Beta and SE in the combined cohort are calculated from the meta-analysis of discovery and replication stages.
(PDF)

S7 Fig Forest plot for the significant association in GTEx hypothalamus. *LRRC37A2* reached transcriptome-wide significance in the TWAS in GTEx hypothalamus. Standardized effect sizes (beta) and SEs are provided for all cohorts. Beta and SE in the discovery cohort are meta-analyzed results based on AGP, SSC, and SPARK. Beta and SE in the combined cohort are calculated from the meta-analysis of discovery and replication stages.
(PDF)

S8 Fig Forest plot for significant associations in GTEx nucleus accumbens basal ganglia. *SLC35G5*, *ARHGAP27*, *LRRC37A2*, *ARL17A*, and *NKX2-2* reached transcriptome-wide significance in the TWAS in GTEx nucleus accumbens basal ganglia. Standardized effect sizes (beta) and SEs are provided for all cohorts. Beta and SE in the discovery cohort are meta-analyzed results based on AGP, SSC, and SPARK. Beta and SE in the combined cohort are calculated from the meta-analysis of discovery and replication stages.
(PDF)

S9 Fig Forest plot for significant associations in GTEx putamen basal ganglia. *SLC35G5* and *LRRC37A2* reached transcriptome-wide significance in the TWAS in GTEx putamen basal ganglia. Standardized effect sizes (beta) and SEs are provided for all cohorts. Beta and SE in the discovery cohort are meta-analyzed results based on AGP, SSC, and SPARK. Beta and SE in the combined cohort are calculated from the meta-analysis of discovery and replication stages.
(PDF)

S10 Fig Forest plot for significant associations in CMC DLPFC. *CTSB*, *DDHD2*, *LOC441455*, *ARHGAP27*, *MAPT*, and *KIZ* reached transcriptome-wide significance in the TWAS in CMC DLPFC. Standardized effect sizes (beta) and SEs are provided for the trio-based cohorts. Beta and SE labeled as the discovery cohort are meta-analyzed results based on AGP, SSC, and SPARK. Effect estimates are not shown in the replication and the combined cohorts since FUSION does not output beta and SE estimates.
(PDF)

S11 Fig Forest plot for significant associations in CMC DLPFC splicing. *SOX7*, *MFHAS1*, *MSRA*, *CRHR1*, *MAPT*, and *XRN2* reached transcriptome-wide significance in the TWAS in CMC DLPFC splicing. Intron cluster IDs are shown below the gene names. Standardized effect sizes (beta) and SEs are provided for the trio-based cohorts. Beta and SE labeled as the discovery cohort are meta-analyzed results based on AGP, SSC, and SPARK. Effect estimates are not shown in the replication and the combined cohorts since FUSION does not output beta and SE estimates.
(PDF)

S12 Fig Imputed expression of *POU3F2* in GTEx hippocampus. The x- and y-axes illustrate the imputed gene expression of *POU3F2* in GTEx hippocampus using hard calls and dosages, respectively.
(PNG)

S13 Fig Power curve for disease association of *POU3F2* in hippocampus with imputation noises. The power curve for the disease association of *POU3F2* with imputation errors is shown. Sigma indicates the standard deviation for the imputation error added to the gene expression.
(PNG)

S14 Fig QQ plot for TWAS with added noise in gene expression values. The QQ plot for TWASs with imputation errors added to gene expressions under different simulation settings. SD indicates the standard variation *σ* of the random imputation errors. A suggestive diagonal line is also added in the background.
(PNG)

S15 Fig Mirrored Manhattan plot for TWAS and GWAS results in 3,245 sibling-parent trios. (A) TWAS results are shown in the upper panel. GWAS associations are shown in the lower panel. The dashed line in the upper panel indicates the cross-tissue transcriptome-wide significance cutoff (p = 4.0E-7) and the dashed line in the lower panel is the genome-wide significance cutoff (p = 5.0E-8). TWAS associations for all 12 tissues are shown. (B) The QQ plot for TWAS associations in 3,245 sibling-parent trios for all 12 tissues.
(PDF)

S16 Fig QQ plot for TWAS in 7,805 proband-parent trios after randomly shuffling the status of probands and pseudo siblings. The QQ plot for TWAS associations in 7,805 proband-parent trios after randomly shuffling the status of probands and pseudo siblings for all 12 tissues.
(PDF)

S17 Fig Power comparisons under different disease prevalence. The power curves under different gene expression effect sizes for different disease prevalence are shown. (A) The power curve under disease prevalence 0.90. Under high prevalence, proband vs parents underperforms relative to pseudo sibling approaches, (B) The power curve under disease prevalence 0.07. Under low prevalence, 1 pseudo sibling underperforms relative to 3 pseudo siblings and parent-proband matching.
(PDF)

S18 Fig Scatterplot of TWAS p-values between different matching methods in hippocampus. (A) The −log_10_ P values between 3-sibling and 1-sibling matching. (B) The −log_10_ P values between 3-sibling and parent-control matching. (C) The QQ plots for 3-sibling, 1-sibling, and parent-control matching.
(PDF)

S19 Fig QQ plot for TWAS using different matching methods on proband-sibling matchings with shuffled disease status. The QQ plot for 3-sibling, 1-sibling, and parent-control matching performed on 7,805 trios in GTEx hippocampus with shuffled disease status. The 1-sibling matching TWAS is conducted on proband-pseudo sibling pairs where the pseudo siblings were constructed using untransmitted parental alleles. The parent-control matching TWAS is conducted on parents versus a random sample from the quad in 3-sibling matching (**Material and Methods**). The association results were obtained using conditional logistic regression.
(PDF)

S20 Fig Additional significant loci identified in TWAS. We identified 31 transcriptome-wide significant associations from 7 independent loci. Four loci with associations that remained significant after correcting for all genes and all tissues are shown in Fig 3 in the main text. (A) Chromosome 3, 48.4 mb (B) Chromosome 8, 38.5 mb (C) Chromosome 9, 99.7 mb. For each locus, the index SNP with the most significant association in GWAS is marked as purple diamond and the color of data points indicates LD of neighboring SNPs with the index SNP. Genes are highlighted in red if they reached transcriptome-wide significance in at least one tissue. The x-axis denotes genome coordinates and the y-axis denotes association p-values in GWAS.
(PNG)

S21 Fig Multi-tissue gene expression profile of *POU3F2* in GTEx Release V8. (PDF)

S22 Fig The spatiotemporal expression pattern of candidate genes identified in TWAS. The spatiotemporal expression pattern of 17 TWAS genes across 5 brain regions and 12 developmental stages. The periods span fetal development, infancy, childhood, adolescence, and adulthood, from 4 post-conceptional weeks (PCW) to 40 postnatal years (Y). The dashed line indicates the boundary between later fetal and early infancy stages (0 month).
(PNG)

S23 Fig Enrichment of *de novo* mutations in 987 non-ASD genes regulated by *POU3F2*. Enrichment results in 2,508 ASD probands and 1,911 unaffected siblings across four annotation categories (all mutations, loss-of-function, missense, deleterious missense, and synonymous) are shown. p-values are shown above each bar.
(PNG)

S1 Table Transcriptome-wide significant associations in TWAS meta-analysis. Beta and SE indicate the standardized effect size and standard error estimates in conditional logistic regression. Some effect size estimates are unavailable in the replication cohort since FUSION does not provide effect size estimates.
(XLSX)

S2 Table Genome-wide significant loci in GWAS meta-analysis. Beta and SE indicate the effect size estimates with respect to A1 counts and standard error in GWAS.
(XLSX)

S3 Table Fine-mapping TWAS results on 7,805 ASD trios. Fine-mapping results on loci with different significant associations. The intron usage clustering ID is listed in the parenthesis, if applicable. P indicates the p-value in multivariate conditional logistic regression.
(XLSX)

S4 Table Gene set enrichment results based on nominally significant TWAS genes (P<0.05). The expected and observed values of gene set overlap are shown. The size of gene set indicates the number of overlapped genes between all genes in the TWAS and the pre-specified gene set. P-values were calculated using hypergeometric test.
(XLSX)

S5 Table Sex-stratified and FSIQ-stratified TWAS results. Beta and SE indicate the standardized effect size and standard error estimates.
(XLSX)

S6 Table Hi-C interaction statistics with *POU3F2* promoter region. P-values and q-values were calculated by Fit-Hi-C. NegLogP and negLogQ are negative log10 transformed p-values and q-values.
(XLSX)

S7 Table TAD regions in CP and GZ zones. (XLSX)

S8 Table Predictive weights in the imputation model for *POU3F2* in GTEx hippocampus. (XLSX)

S9 Table Predicted target genes of *POU3F2*. (XLSX)

S10 Table Coexpression between ASD genes regulated by *POU3F2* and *POU3F2* in Hippocampus. P indicates the P-value for coexpression.
(XLSX)

S11 Table *De novo* mutation enrichment in *POU3F2* target genes. The observed and expected mutation counts in each annotation category are shown. P-values were calculated using the Poisson test.
(XLSX)

S12 Table *De novo* mutation enrichment in *POU3F*2 target genes, after removing genes in the SPARK gene list or with SFARI scores S-2. The observed and expected mutation counts in each annotation category are shown. P-values were calculated using the Poisson test.
(XLSX)

S13 Table Enrichment of ASD heritability in *POU3F2* binding sites. (XLSX)

S14 Table TWAS association summary statistics for *POU3F2* in GTEx brain regions. Z and P indicate the Z statistics and p-values. R2 indicates the expression imputation qualities in UTMOST training dataset, measured by the square of correlation coefficients between true and predicted gene expressions. Some R2 values are unavailable due to all zero predicted gene expression from low sample sizes during training.
(XLSX)
