# Supplementary figures and images for: Transcriptome-wide transmission disequilibrium analysis identifies novel risk genes for autism spectrum disorder
Source: PLoS Genet. 2021 Feb 4;17(2):e1009309. doi: 10.1371/journal.pgen.1009309 (PMC7888619; doi:10.1371/journal.pgen.1009309)

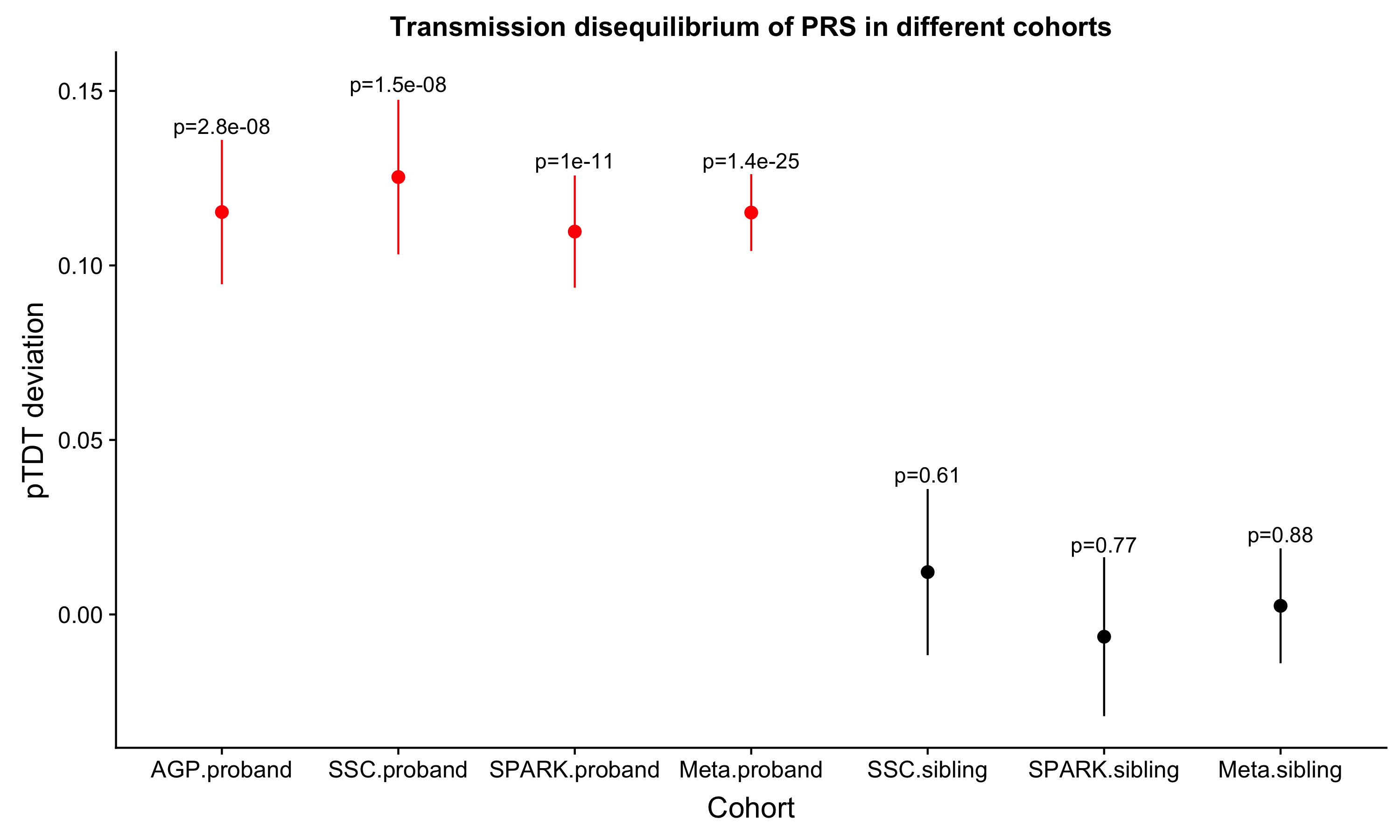

Supplement: S1 Fig — Transmission disequilibrium was quantified by the pTDT approach. Results in probands and unaffected siblings are highlighted in different colors. The mean pTDT deviation and the SE are shown. P-values are labeled above each interval. (PNG) [file pgen.1009309.s001.png]

# ***LRRC37A2***

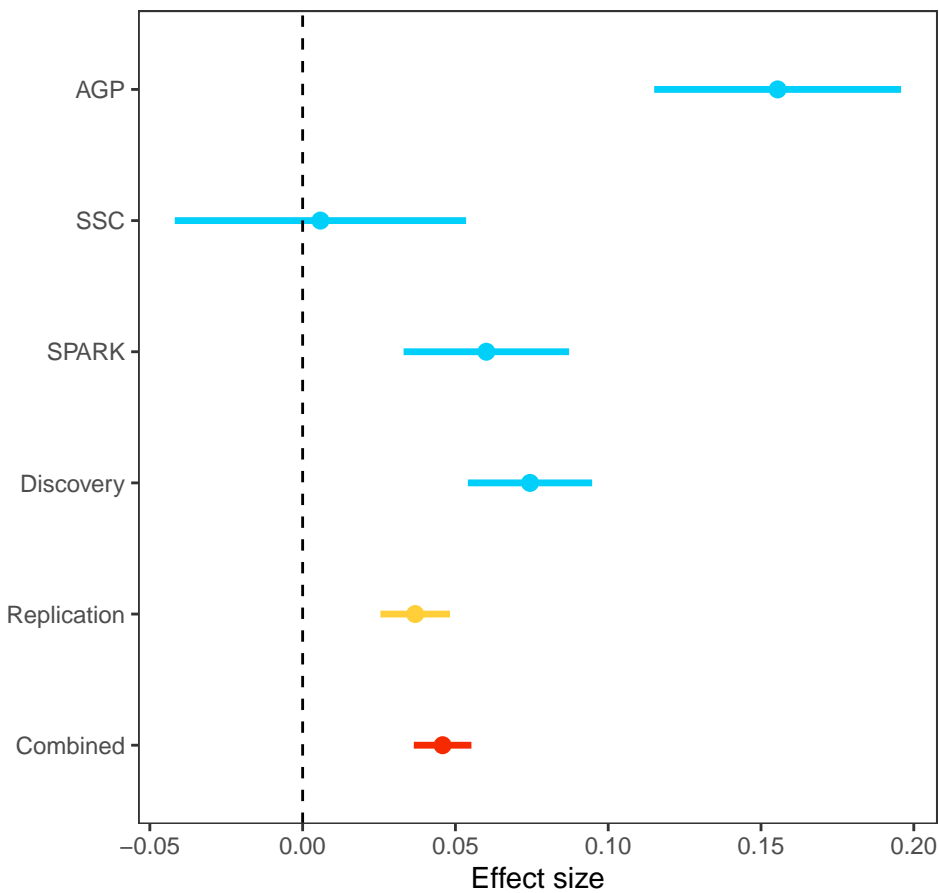

Supplement: S2 Fig — LRRC37A2 reached transcriptome-wide significance in the TWAS in GTEx anterior cingulate cortex BA24. Standardized effect sizes (beta) and SEs are provided for all cohorts. Beta and SE in the discovery cohort are meta-analyzed results based on AGP, SSC, and SPARK. Beta and SE in the combined cohort are calculated from the meta-analysis of discovery and replication stages. (PDF) [file pgen.1009309.s002.pdf]

**FBXW12**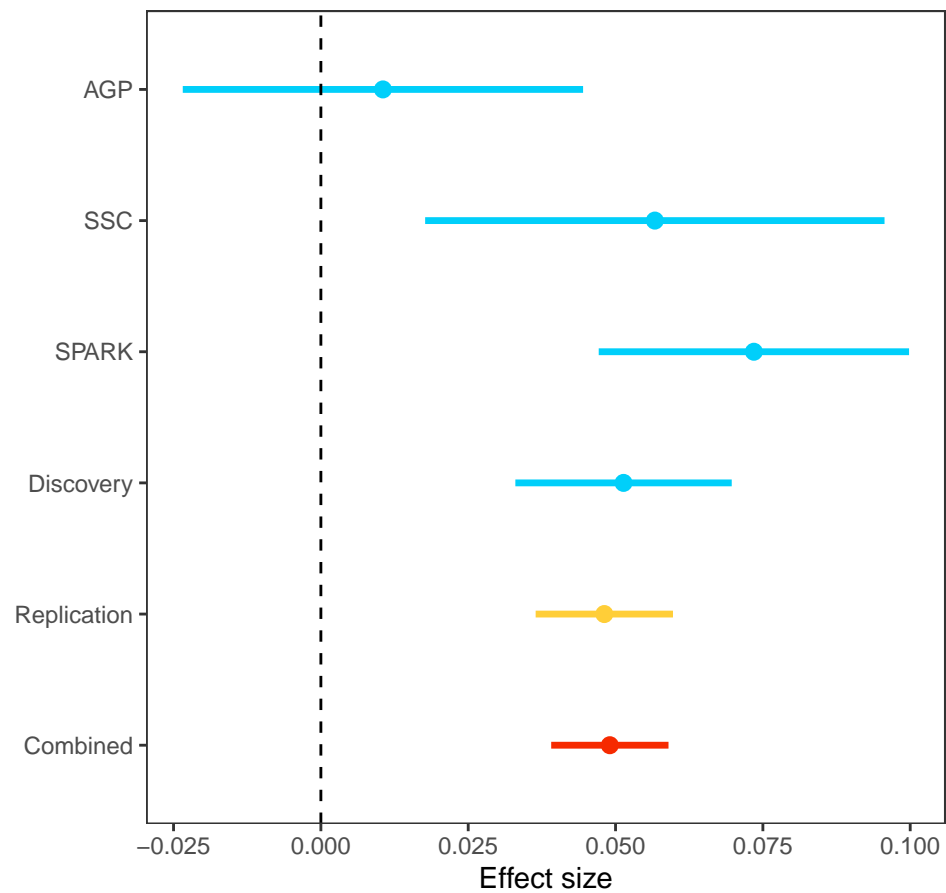**LRRC37A2**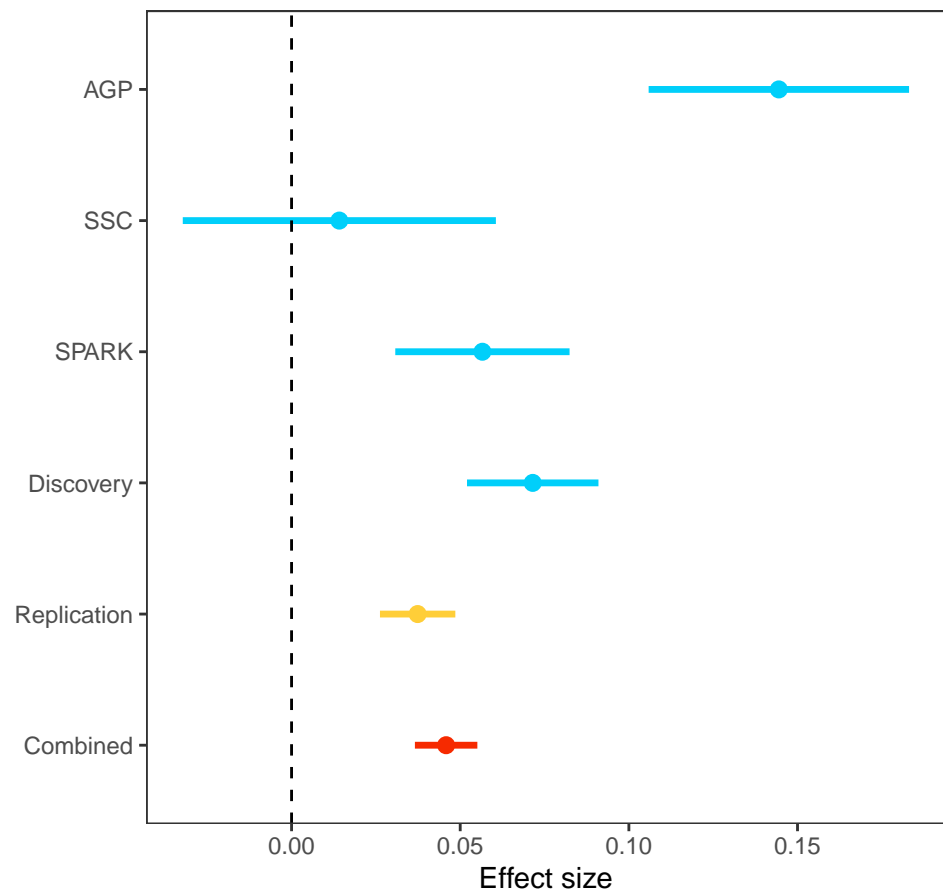

Supplement: S3 Fig — FBXW12 and LRRC37A2 reached transcriptome-wide significance in the TWAS in GTEx caudate basal ganglia. Standardized effect sizes (beta) and SEs are provided for all cohorts. Beta and SE in the discovery cohort are meta-analyzed results based on AGP, SSC, and SPARK. Beta and SE in the combined cohort are calculated from the meta-analysis of discovery and replication stages. (PDF) [file pgen.1009309.s003.pdf]

**NME6**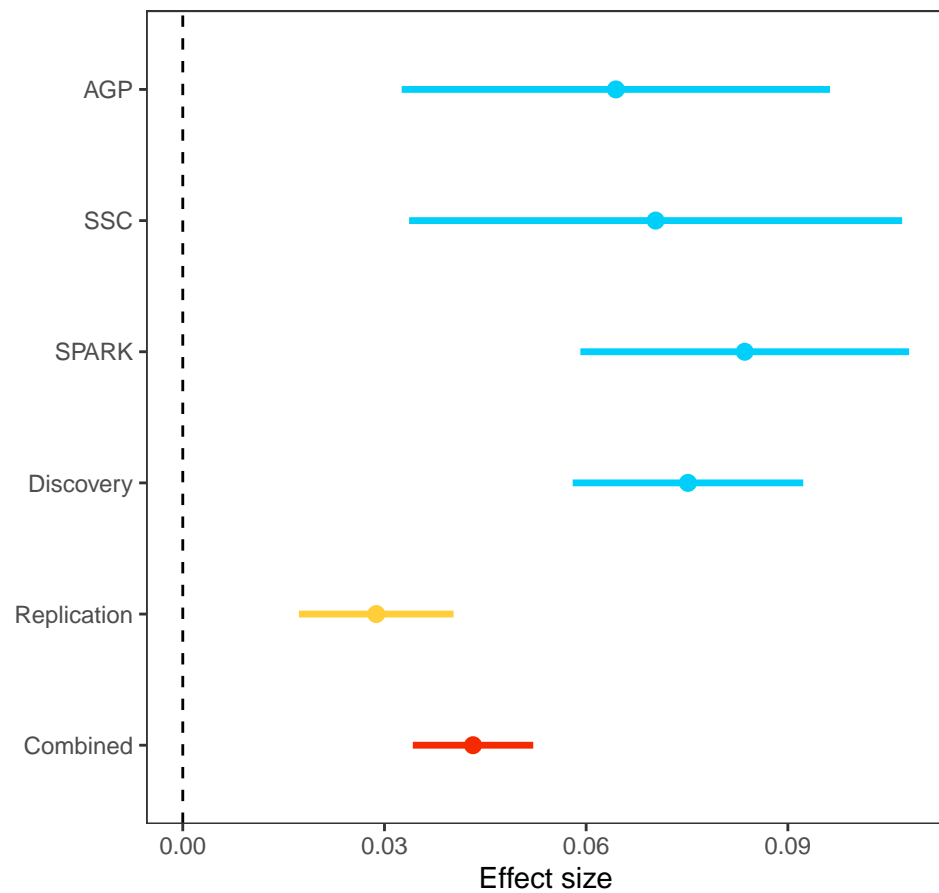**LRRC37A2**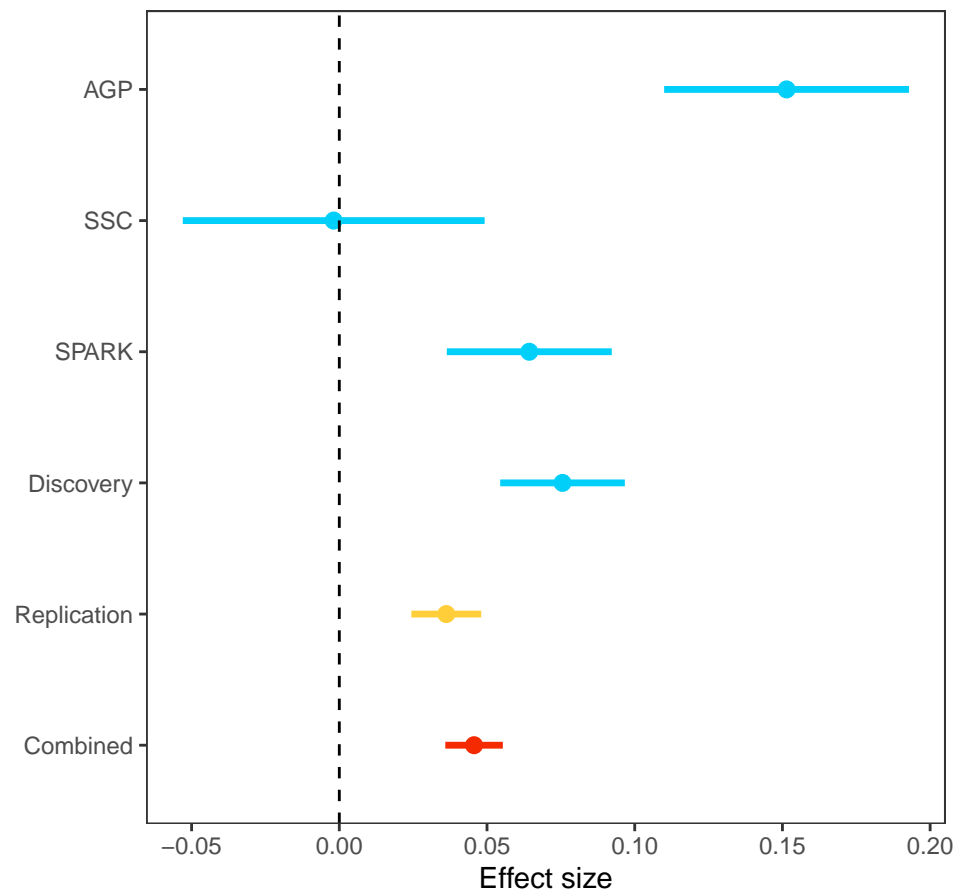

Supplement: S4 Fig — NME6 and LRRC37A2 reached transcriptome-wide significance in the TWAS in GTEx cerebellar hemisphere. Standardized effect sizes (beta) and SEs are provided for all cohorts. Beta and SE in the discovery cohort are meta-analyzed results based on AGP, SSC, and SPARK. Beta and SE in the combined cohort are calculated from the meta-analysis of discovery and replication stages. (PDF) [file pgen.1009309.s004.pdf]

***MAPT***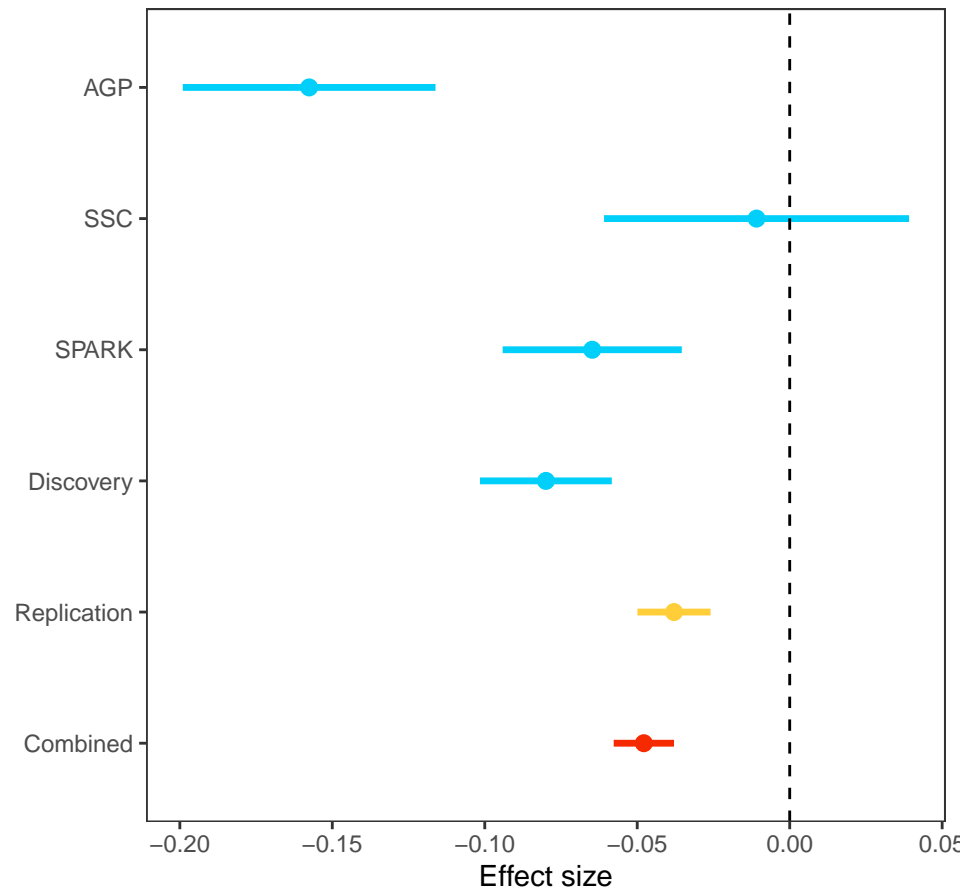***LRRC37A2***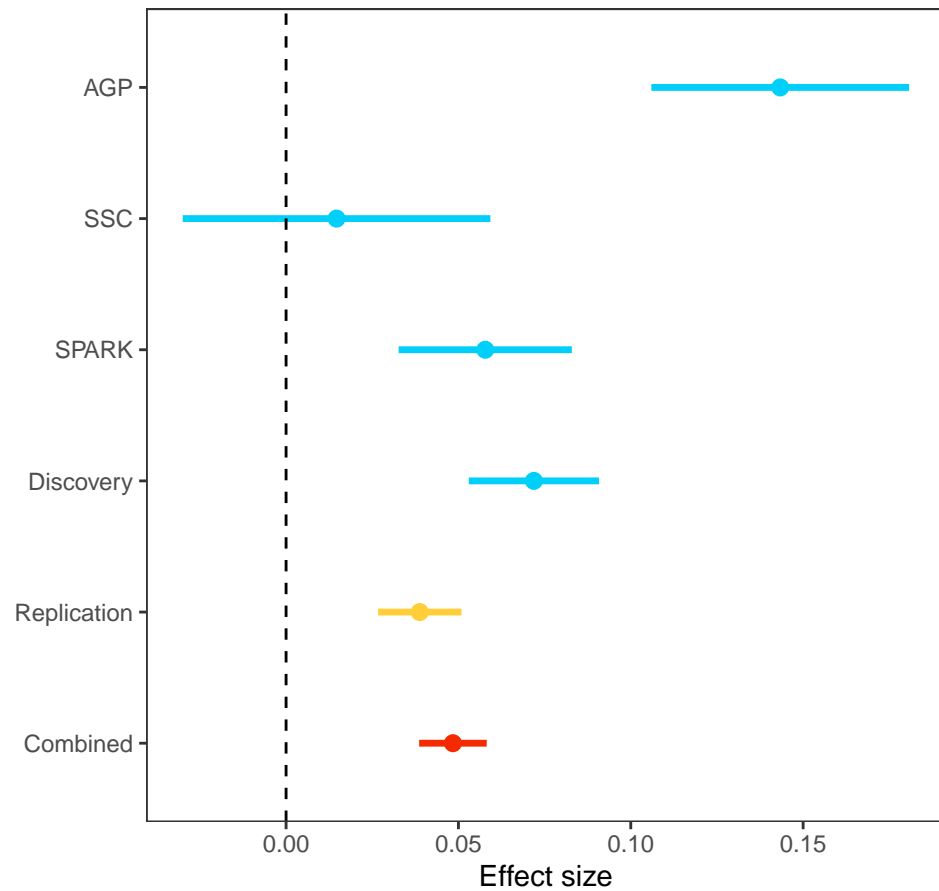

Supplement: S5 Fig — MAPT and LRRC37A2 reached transcriptome-wide significance in the TWAS in GTEx cerebellum. Standardized effect sizes (beta) and SEs are provided for all cohorts. Beta and SE in the discovery cohort are meta-analyzed results based on AGP, SSC, and SPARK. Beta and SE in the combined cohort are calculated from the meta-analysis of discovery and replication stages. (PDF) [file pgen.1009309.s005.pdf]

# ***POU3F2***

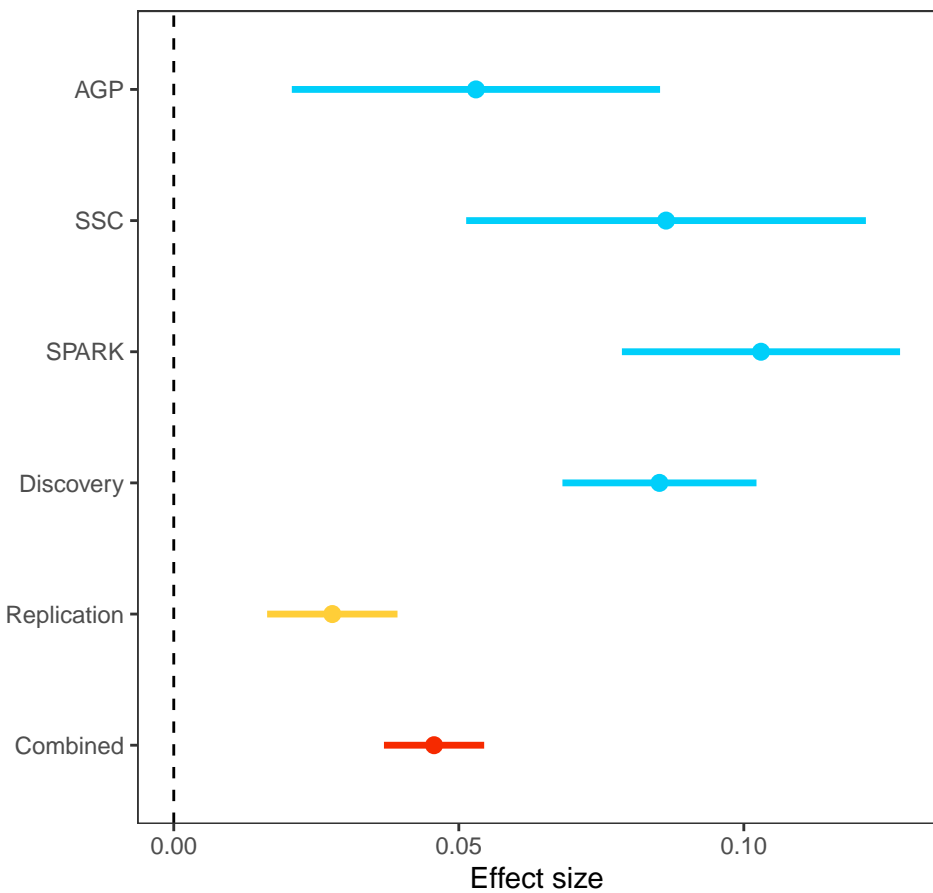

Supplement: S6 Fig — POU3F2 reached transcriptome-wide significance in the TWAS in GTEx hippocampus. Standardized effect sizes (beta) and SEs are provided for all cohorts. Beta and SE in the discovery cohort are meta-analyzed results based on AGP, SSC, and SPARK. Beta and SE in the combined cohort are calculated from the meta-analysis of discovery and replication stages. (PDF) [file pgen.1009309.s006.pdf]

# ***LRRC37A2***

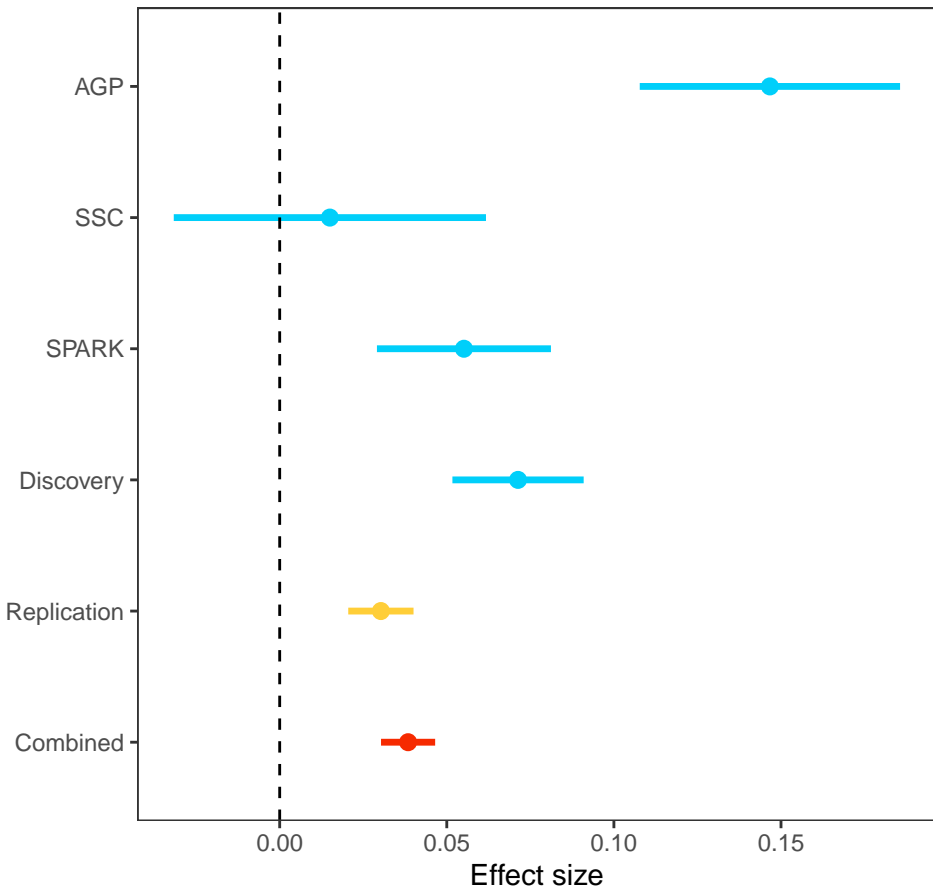

Supplement: S7 Fig — LRRC37A2 reached transcriptome-wide significance in the TWAS in GTEx hypothalamus. Standardized effect sizes (beta) and SEs are provided for all cohorts. Beta and SE in the discovery cohort are meta-analyzed results based on AGP, SSC, and SPARK. Beta and SE in the combined cohort are calculated from the meta-analysis of discovery and replication stages. (PDF) [file pgen.1009309.s007.pdf]

**SLC35G5**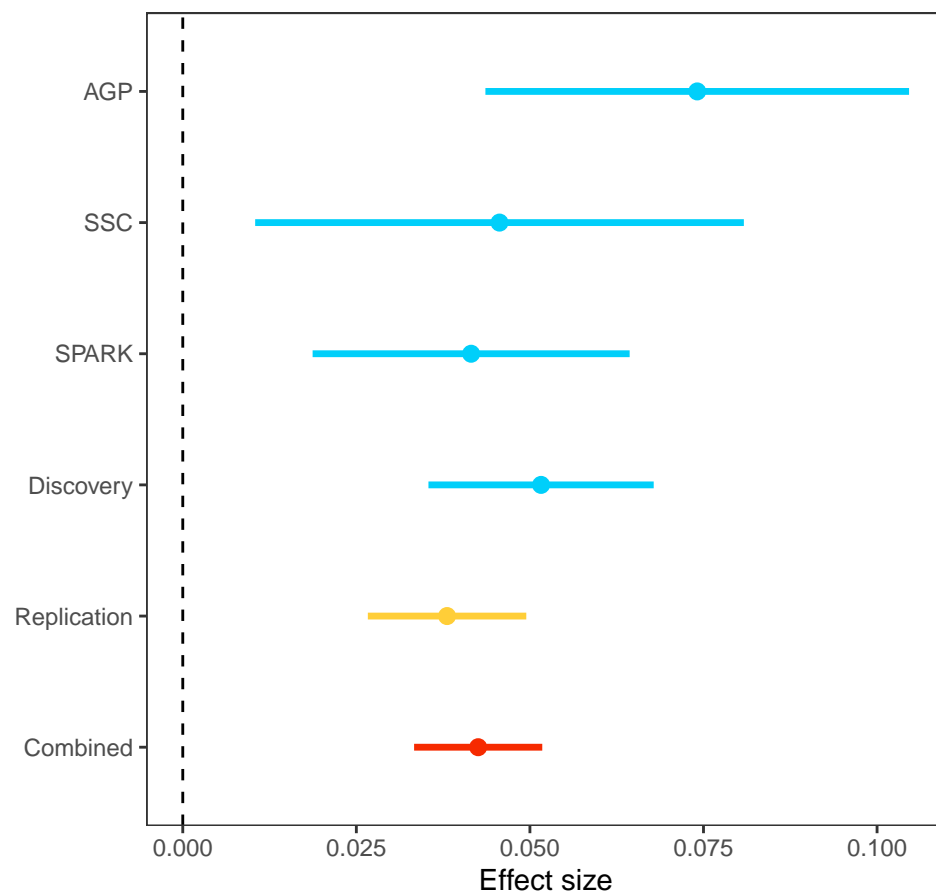**ARHGAP27**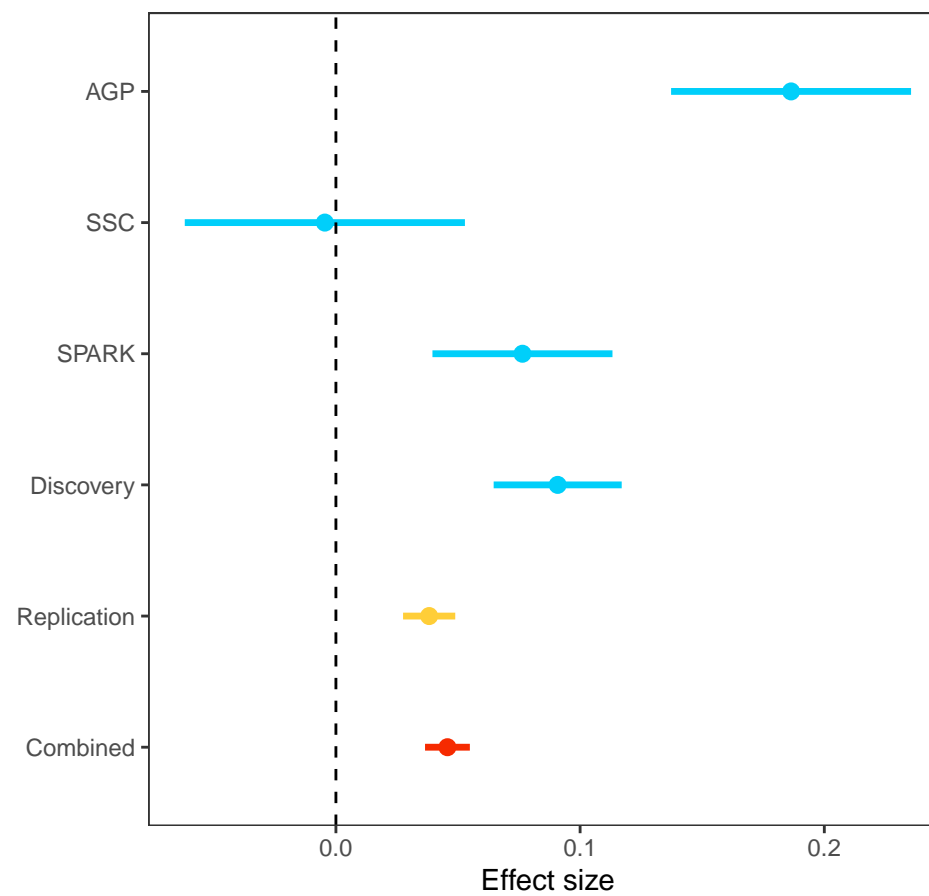**LRRC37A2**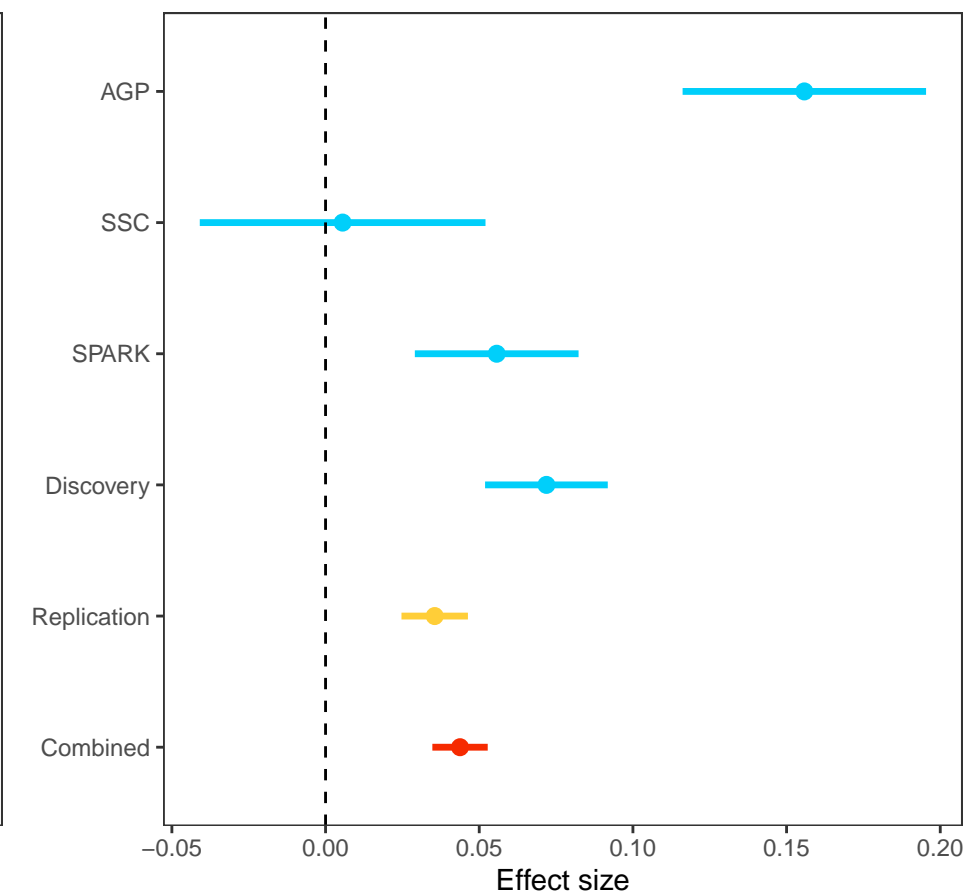**ARL17A**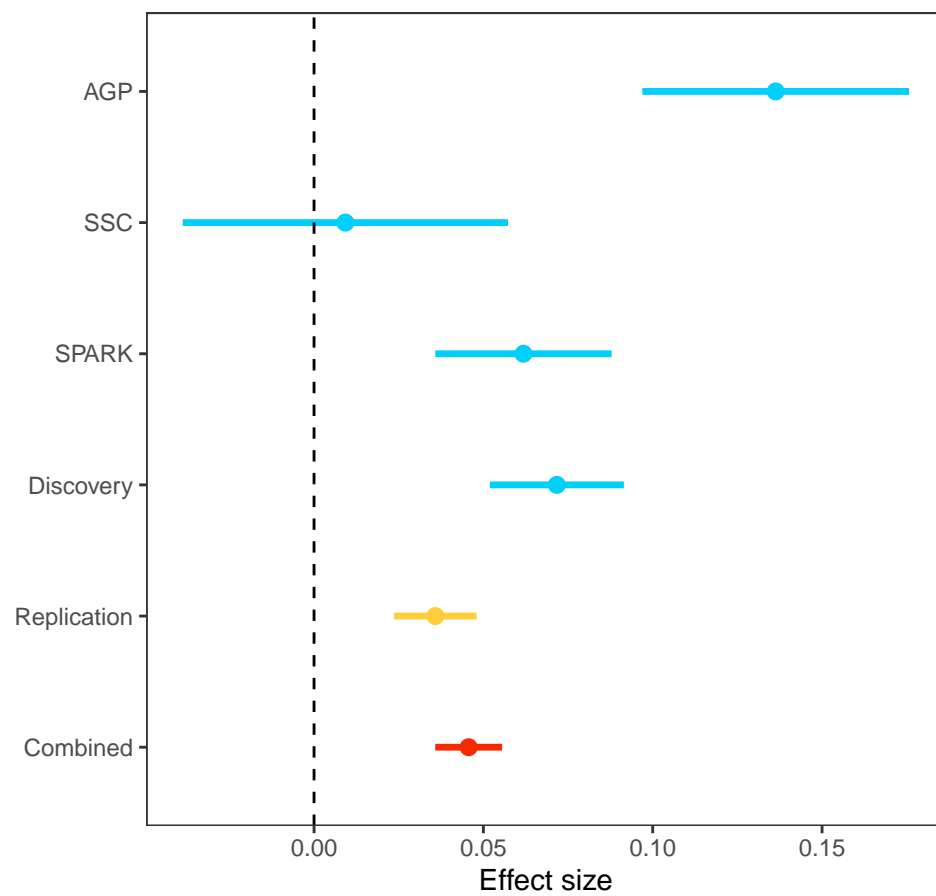**NKX2-2**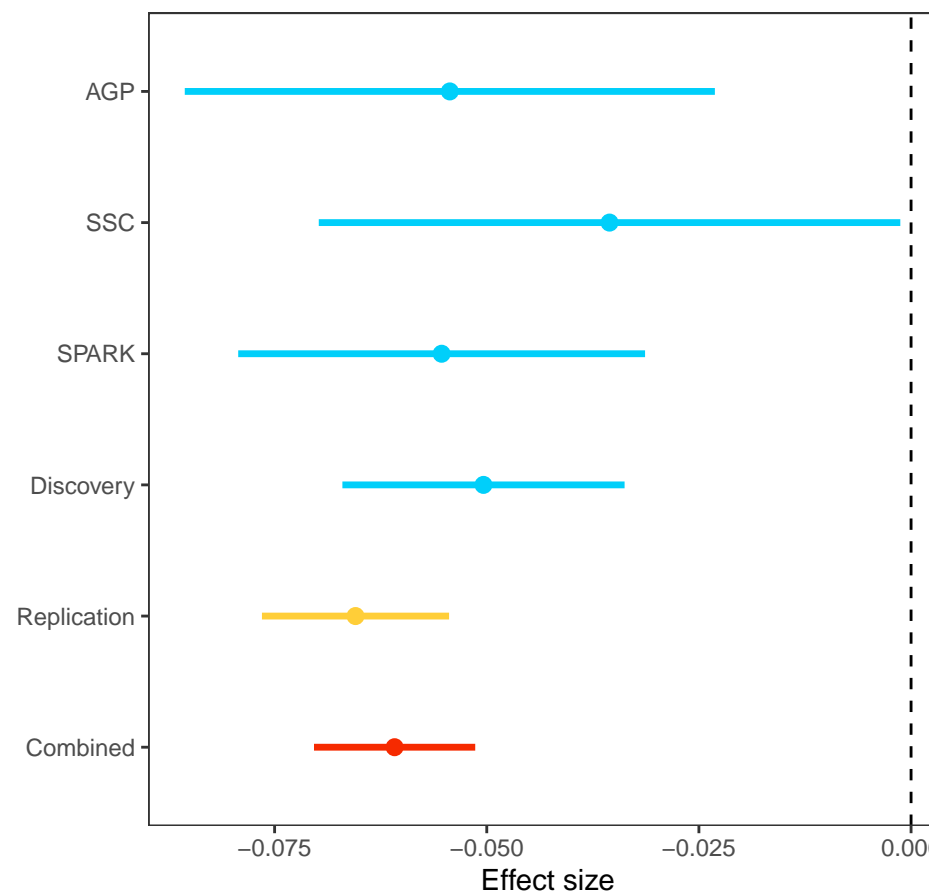

Supplement: S8 Fig — SLC35G5, ARHGAP27, LRRC37A2, ARL17A, and NKX2-2 reached transcriptome-wide significance in the TWAS in GTEx nucleus accumbens basal ganglia. Standardized effect sizes (beta) and SEs are provided for all cohorts. Beta and SE in the discovery cohort are meta-analyzed results based on AGP, SSC, and SPARK. Beta and SE in the combined cohort are calculated from the meta-analysis of discovery and replication stages. (PDF) [file pgen.1009309.s008.pdf]

**SLC35G5**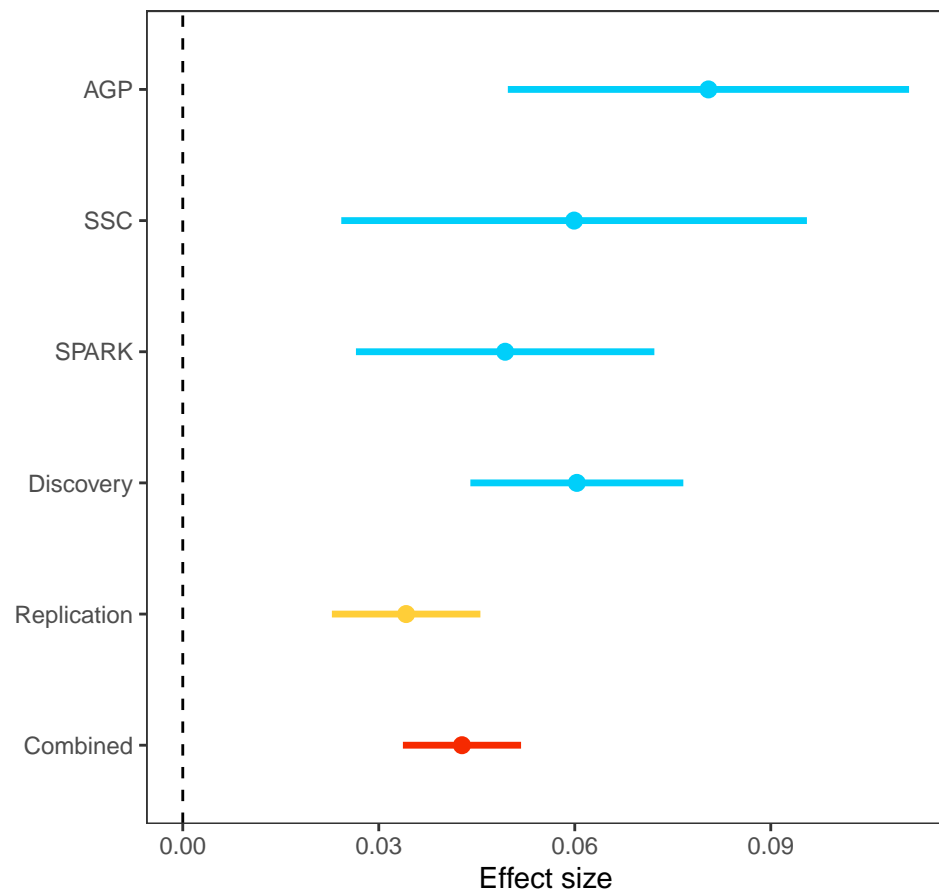**LRRC37A2**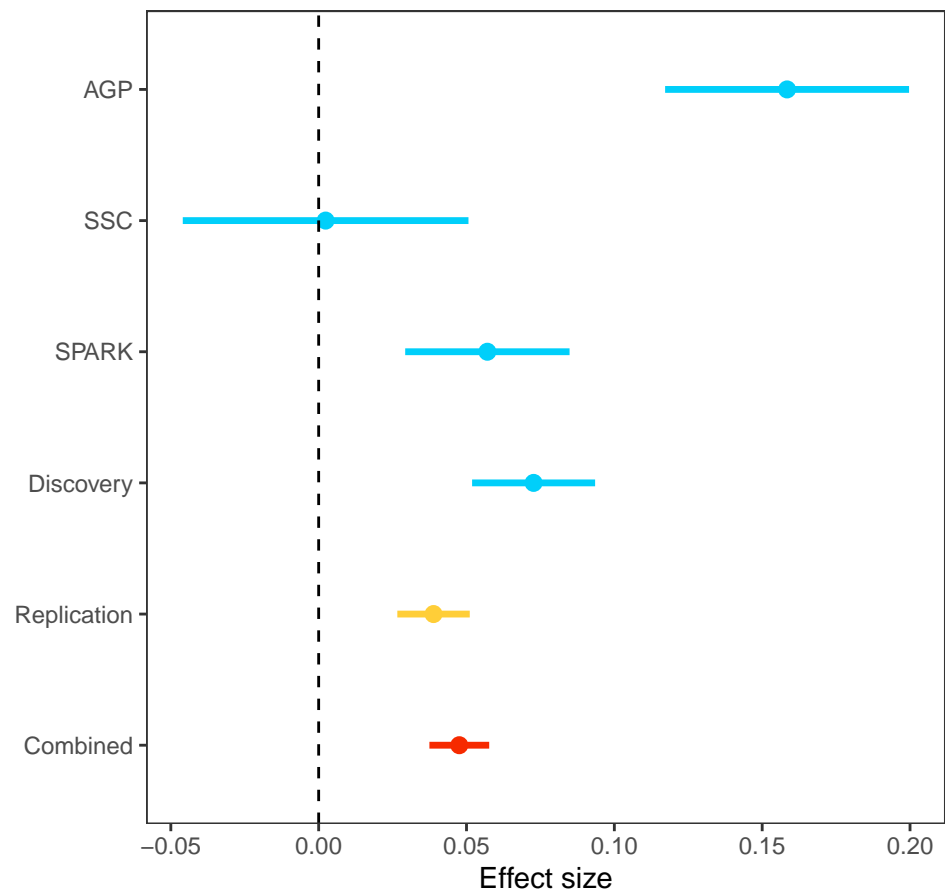

Supplement: S9 Fig — SLC35G5 and LRRC37A2 reached transcriptome-wide significance in the TWAS in GTEx putamen basal ganglia. Standardized effect sizes (beta) and SEs are provided for all cohorts. Beta and SE in the discovery cohort are meta-analyzed results based on AGP, SSC, and SPARK. Beta and SE in the combined cohort are calculated from the meta-analysis of discovery and replication stages. (PDF) [file pgen.1009309.s009.pdf]

**CTSB**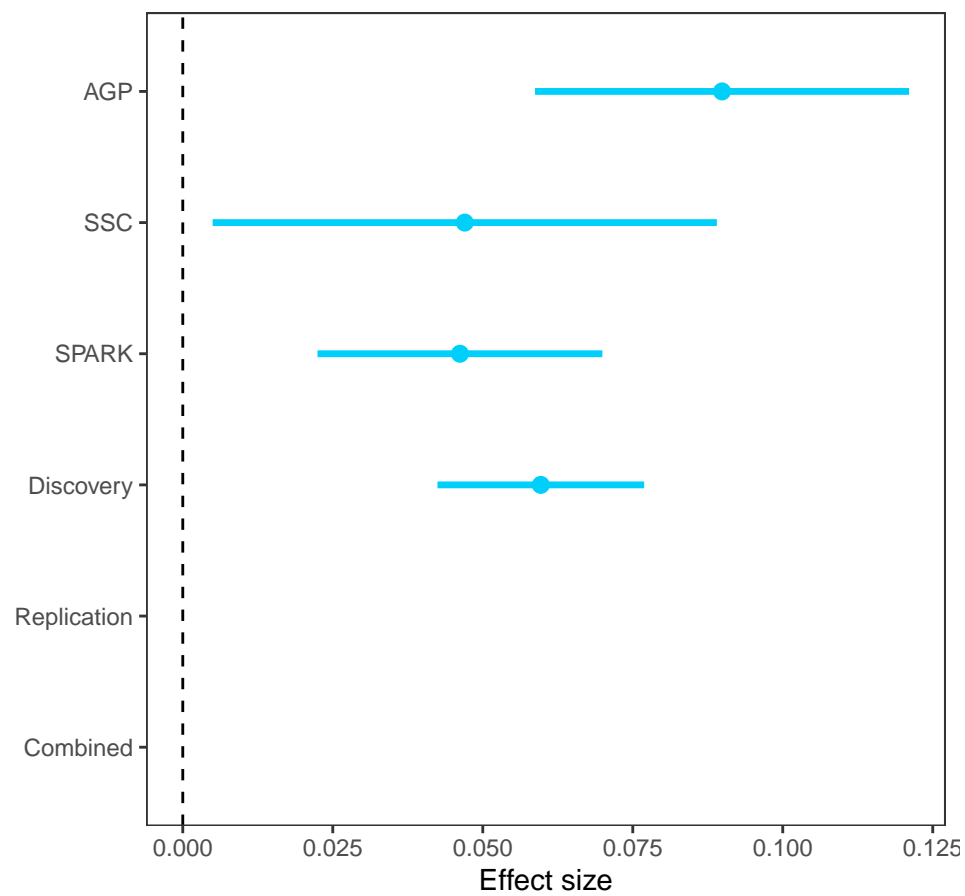**DDHD2**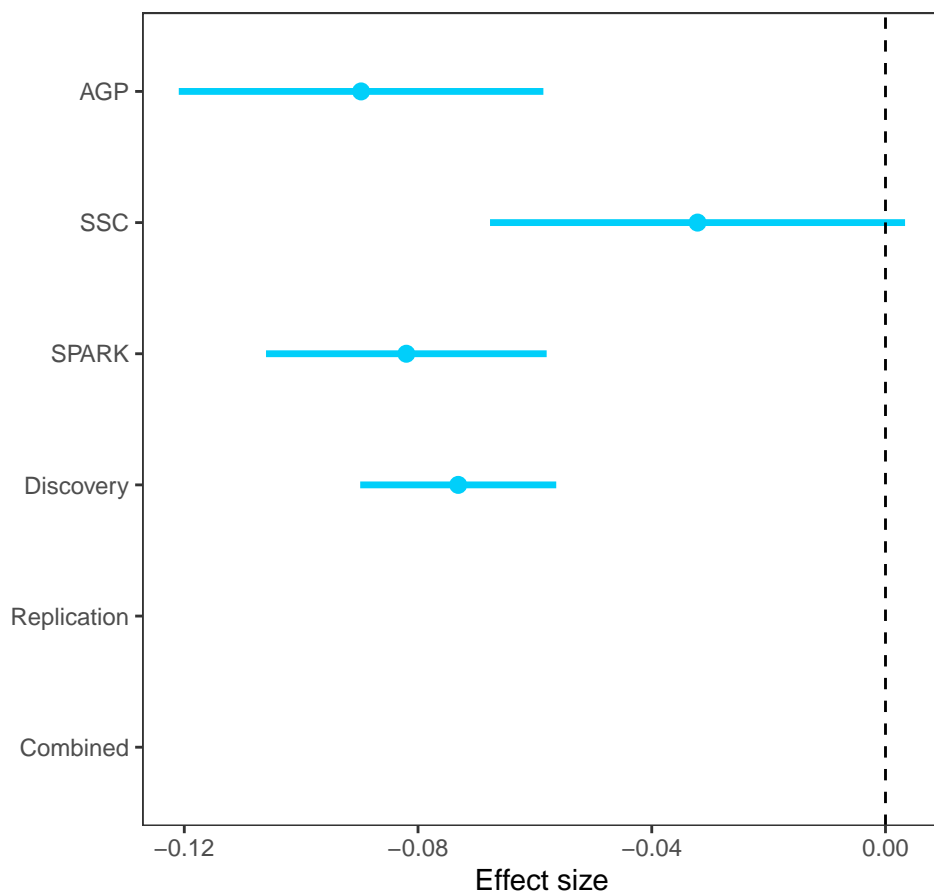**LOC441455**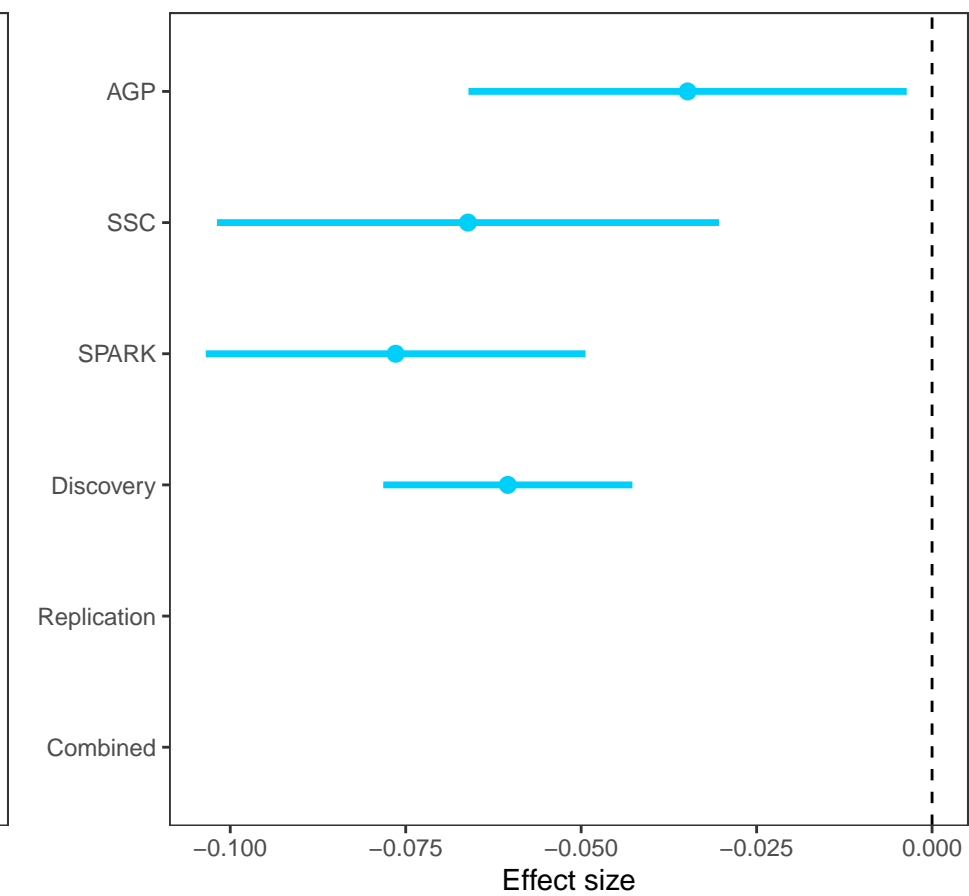**ARHGAP27**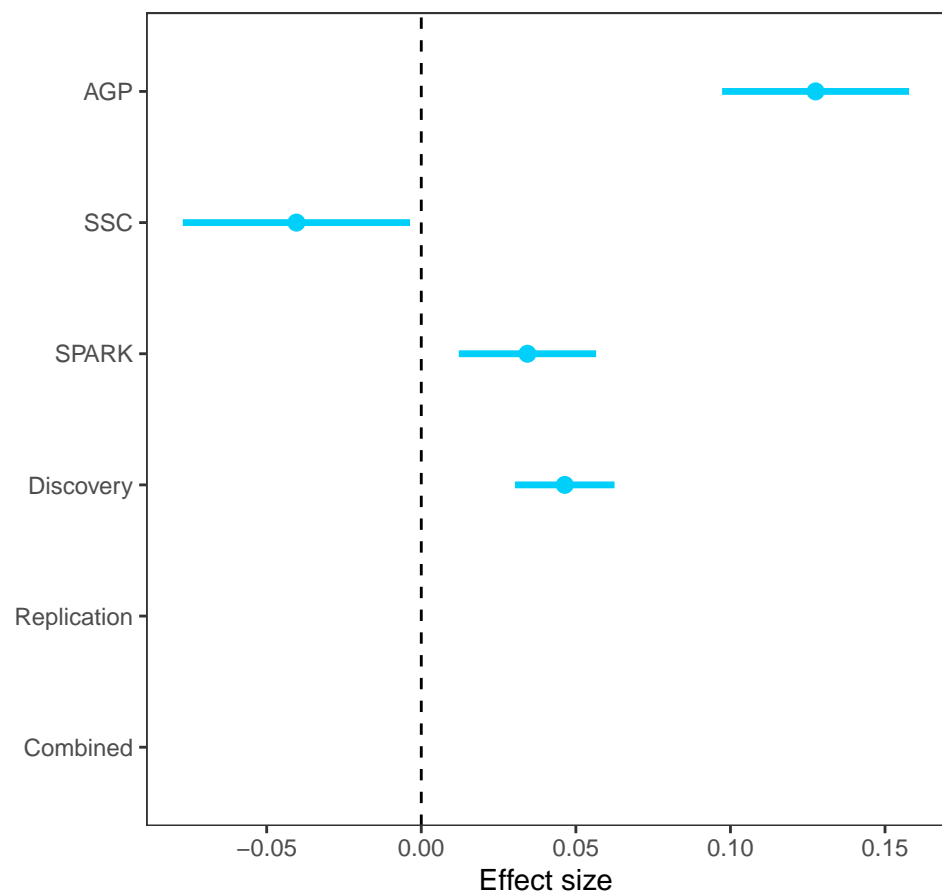**MAPT**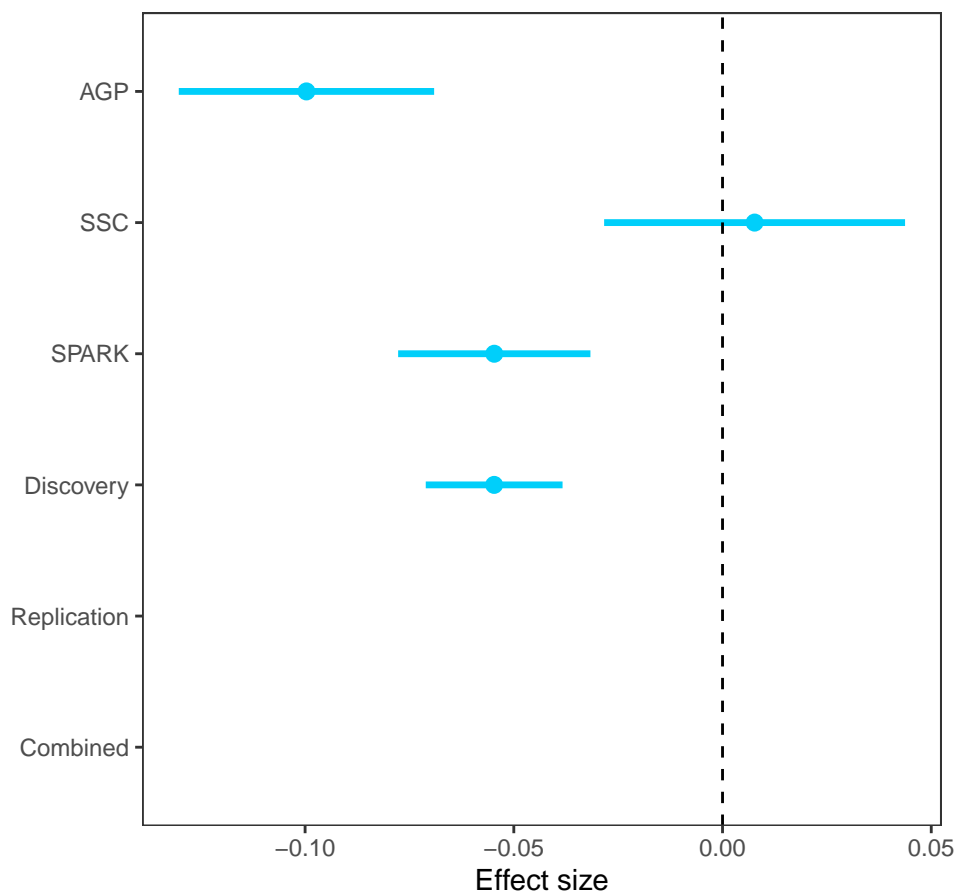**KIZ**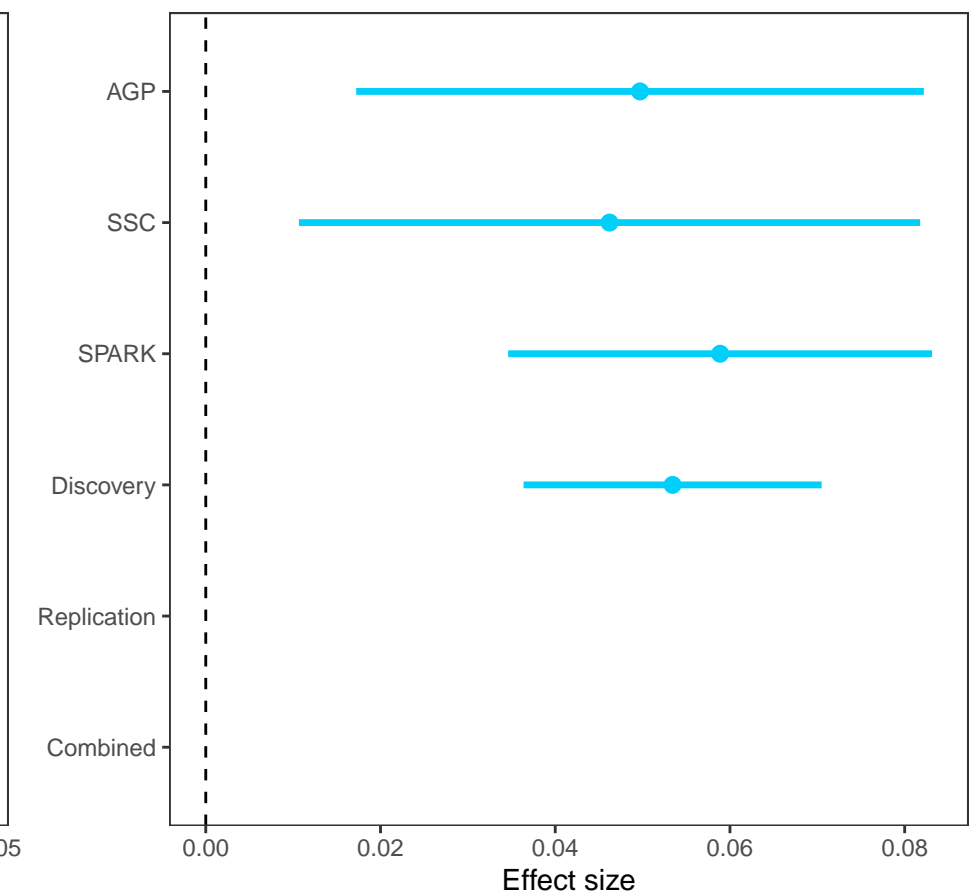

Supplement: S10 Fig — CTSB, DDHD2, LOC441455, ARHGAP27, MAPT, and KIZ reached transcriptome-wide significance in the TWAS in CMC DLPFC. Standardized effect sizes (beta) and SEs are provided for the trio-based cohorts. Beta and SE labeled as the discovery cohort are meta-analyzed results based on AGP, SSC, and SPARK. Effect estimates are not shown in the replication and the combined cohorts since FUSION does not output beta and SE estimates. (PDF) [file pgen.1009309.s010.pdf]

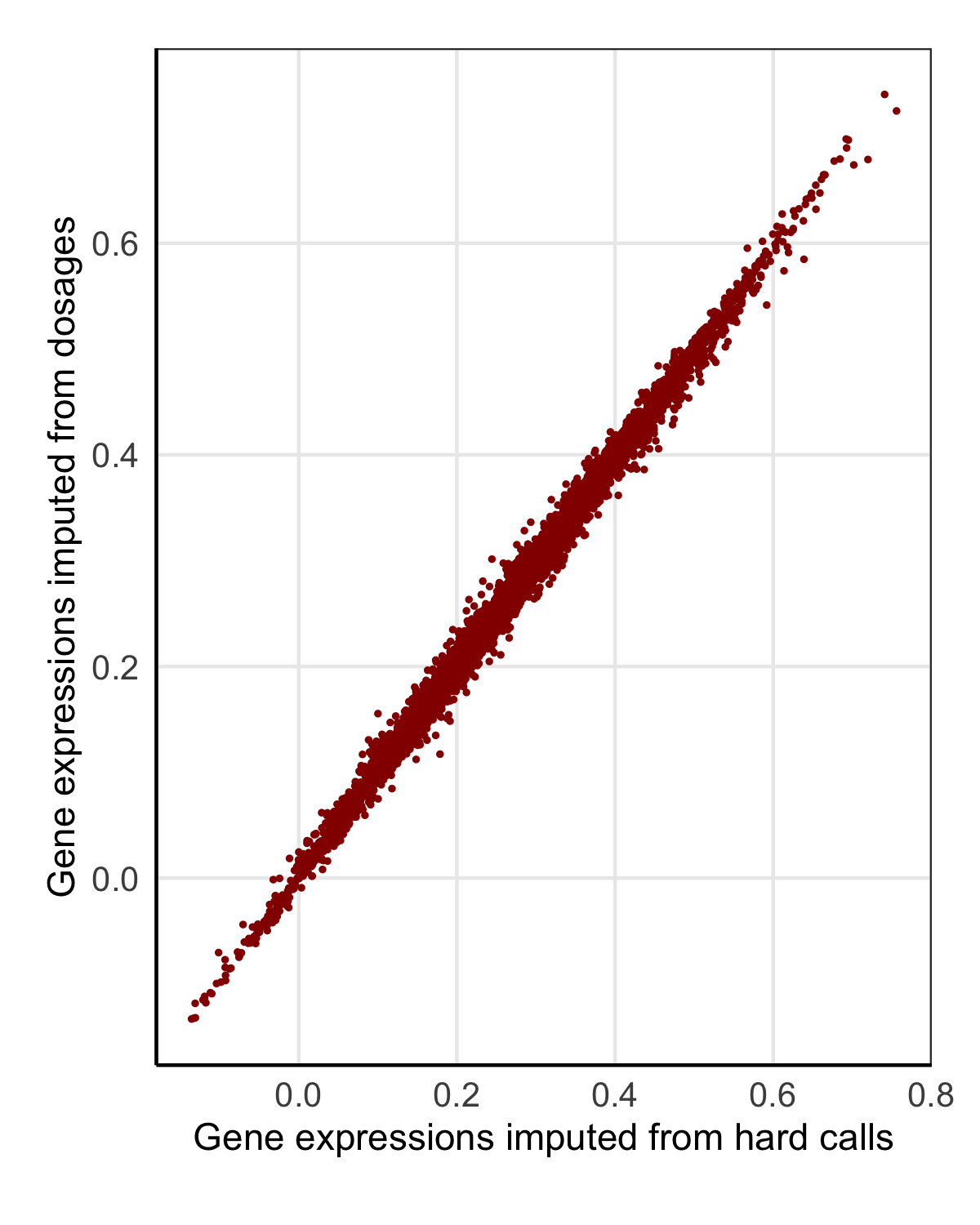

Supplement: S12 Fig — The x- and y-axes illustrate the imputed gene expression of POU3F2 in GTEx hippocampus using hard calls and dosages, respectively. (PNG) [file pgen.1009309.s012.png]

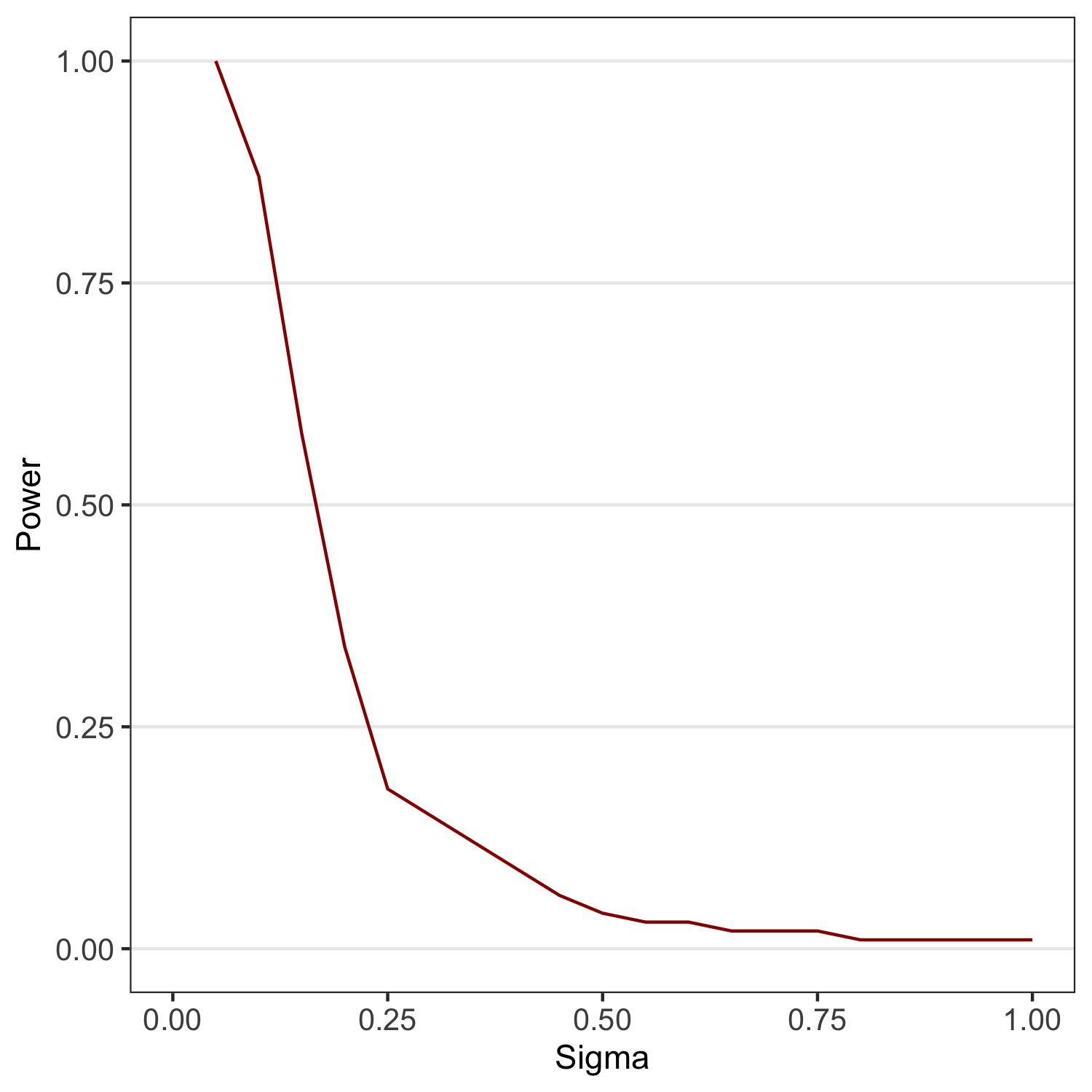

Supplement: S13 Fig — The power curve for the disease association of POU3F2 with imputation errors is shown. Sigma indicates the standard deviation for the imputation error added to the gene expression. (PNG) [file pgen.1009309.s013.png]

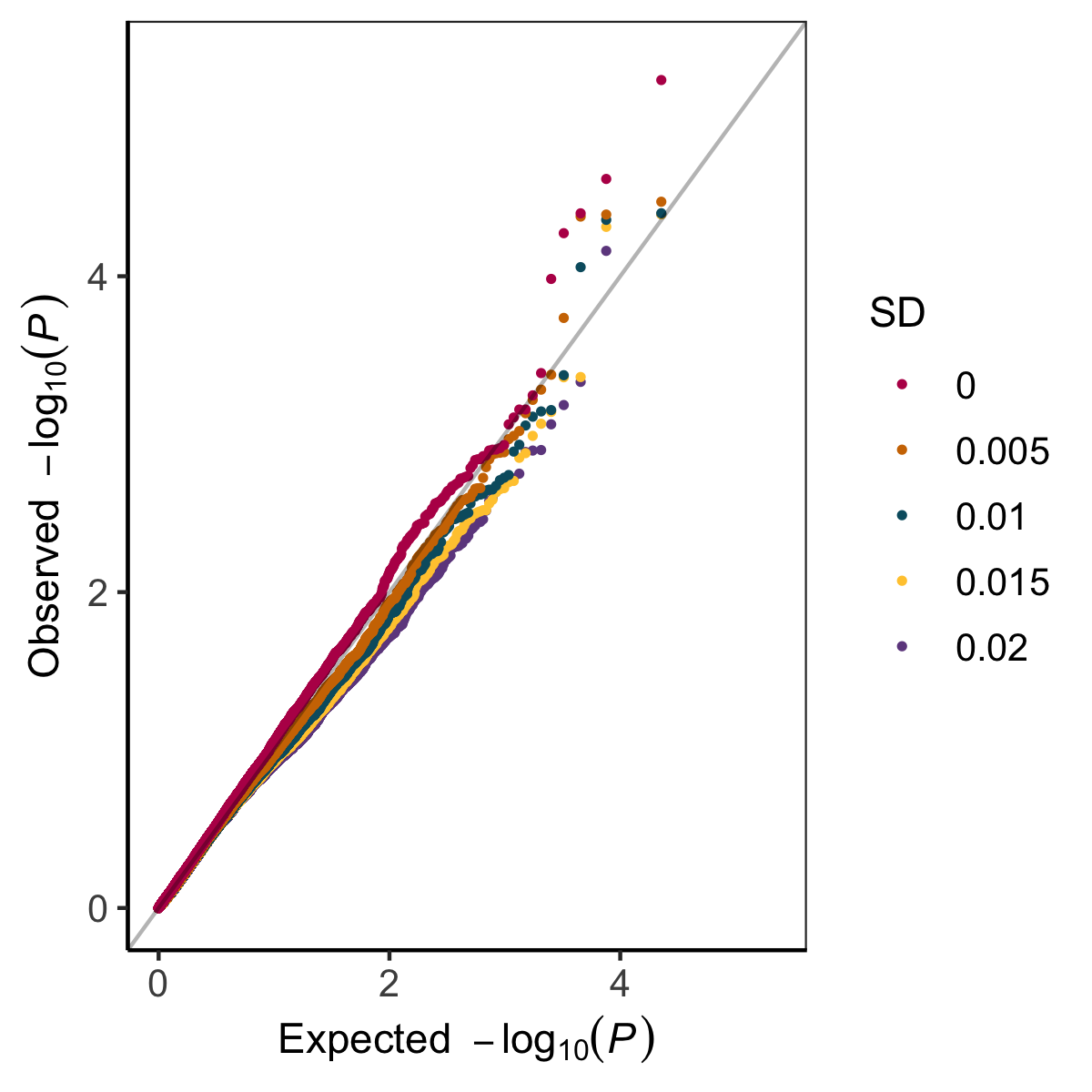

Supplement: S14 Fig — The QQ plot for TWASs with imputation errors added to gene expressions under different simulation settings. SD indicates the standard variation σ of the random imputation errors. A suggestive diagonal line is also added in the background. (PNG) [file pgen.1009309.s014.png]

A

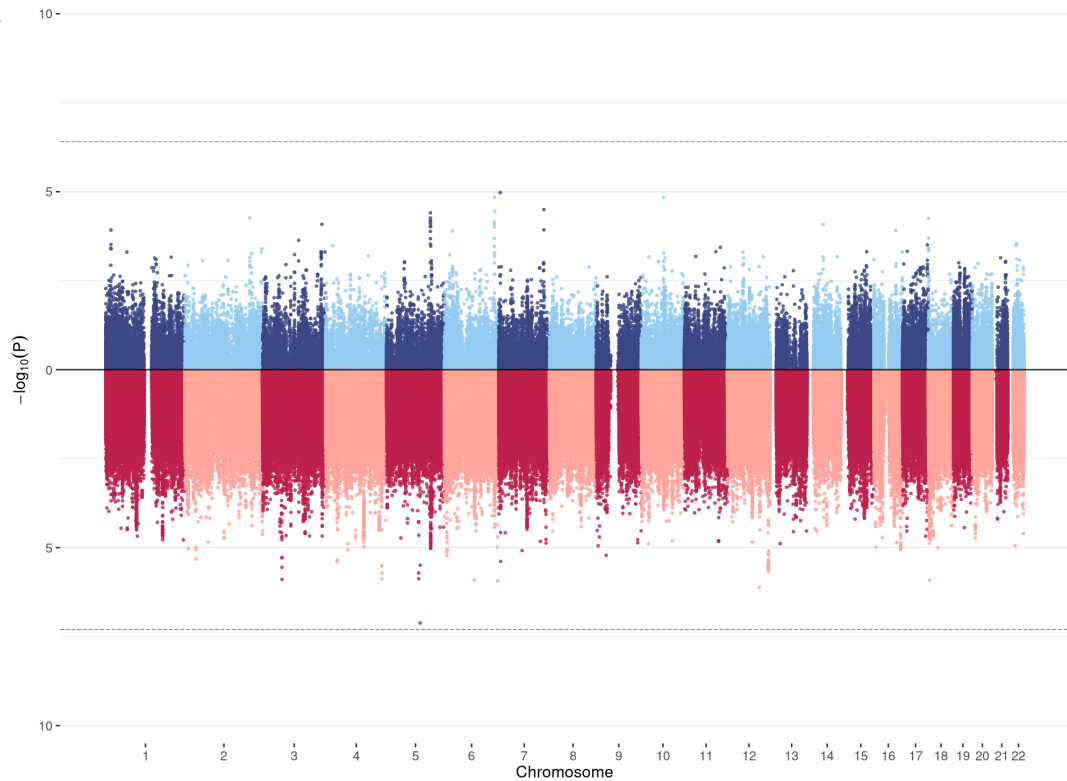

B

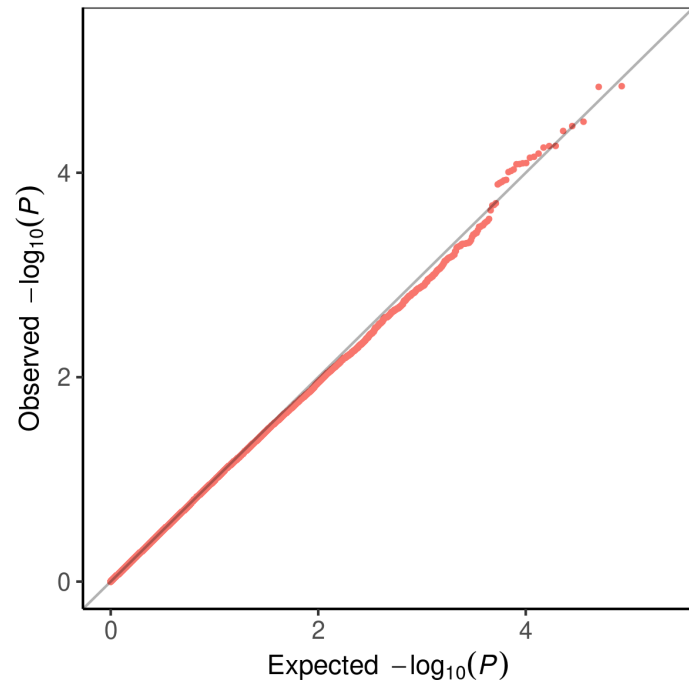

Supplement: S15 Fig — (A) TWAS results are shown in the upper panel. GWAS associations are shown in the lower panel. The dashed line in the upper panel indicates the cross-tissue transcriptome-wide significance cutoff (p = 4.0E-7) and the dashed line in the lower panel is the genome-wide significance cutoff (p = 5.0E-8). TWAS associations for all 12 tissues are shown. (B) The QQ plot for TWAS associations in 3,245 sibling-parent trios for all 12 tissues. (PDF) [file pgen.1009309.s015.pdf]

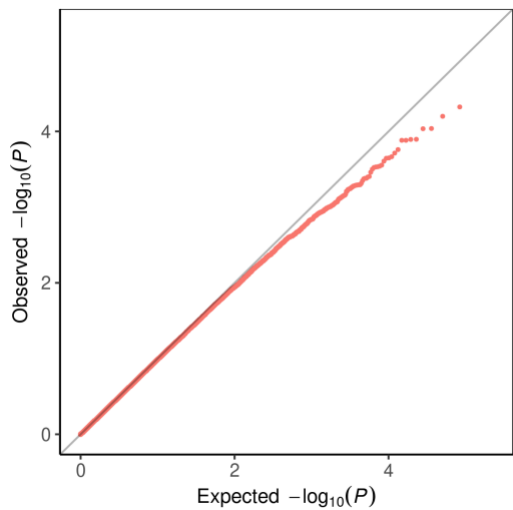

Supplement: S16 Fig — The QQ plot for TWAS associations in 7,805 proband-parent trios after randomly shuffling the status of probands and pseudo siblings for all 12 tissues. (PDF) [file pgen.1009309.s016.pdf]

**A**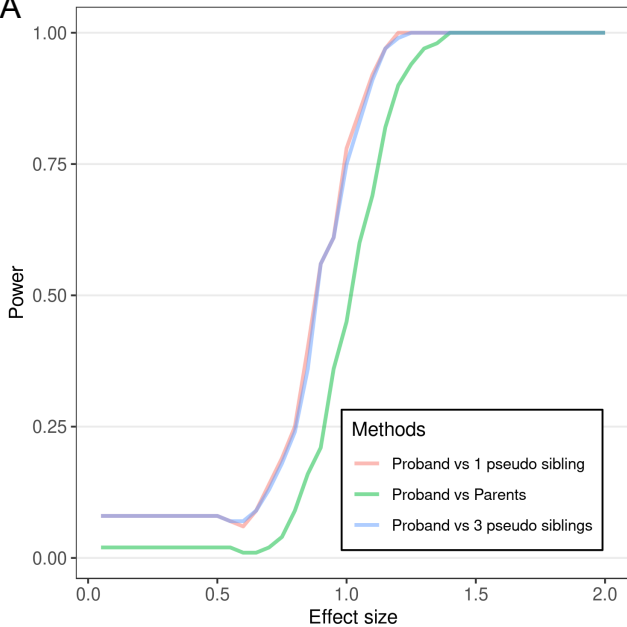**B**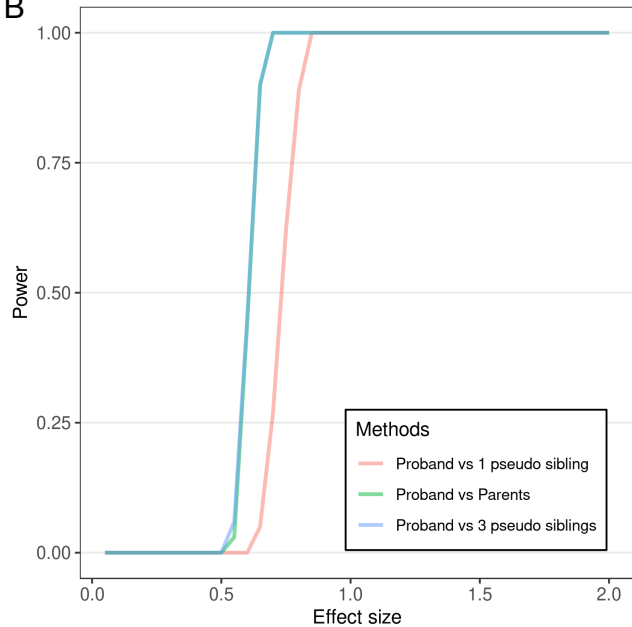

Supplement: S17 Fig — The power curves under different gene expression effect sizes for different disease prevalence are shown. (A) The power curve under disease prevalence 0.90. Under high prevalence, proband vs parents underperforms relative to pseudo sibling approaches, (B) The power curve under disease prevalence 0.07. Under low prevalence, 1 pseudo sibling underperforms relative to 3 pseudo siblings and parent-proband matching. (PDF) [file pgen.1009309.s017.pdf]

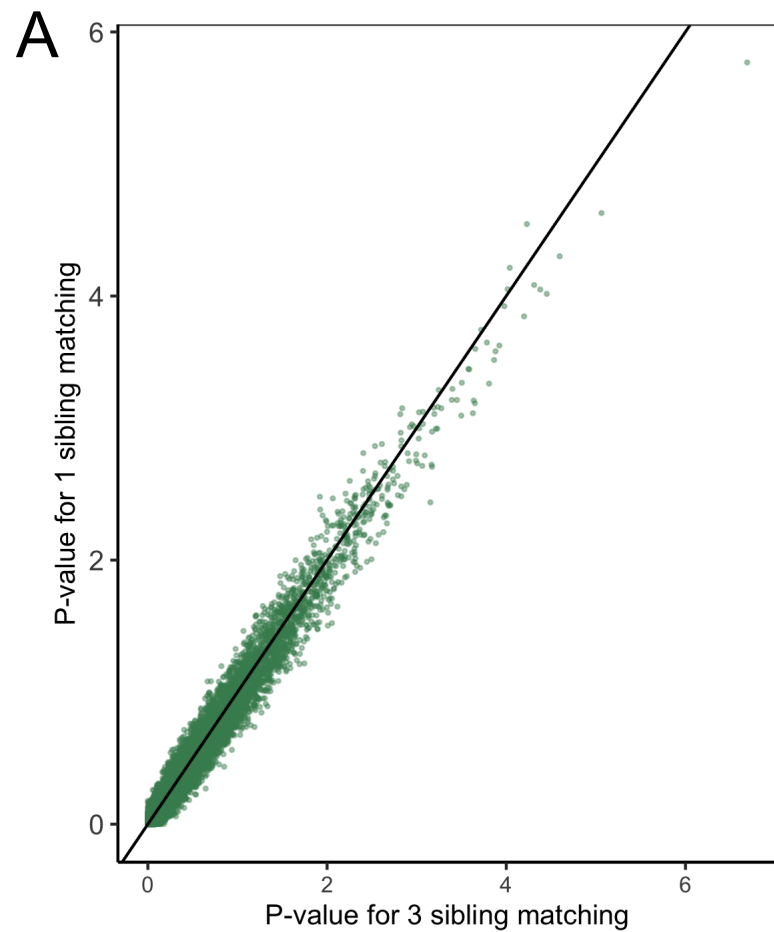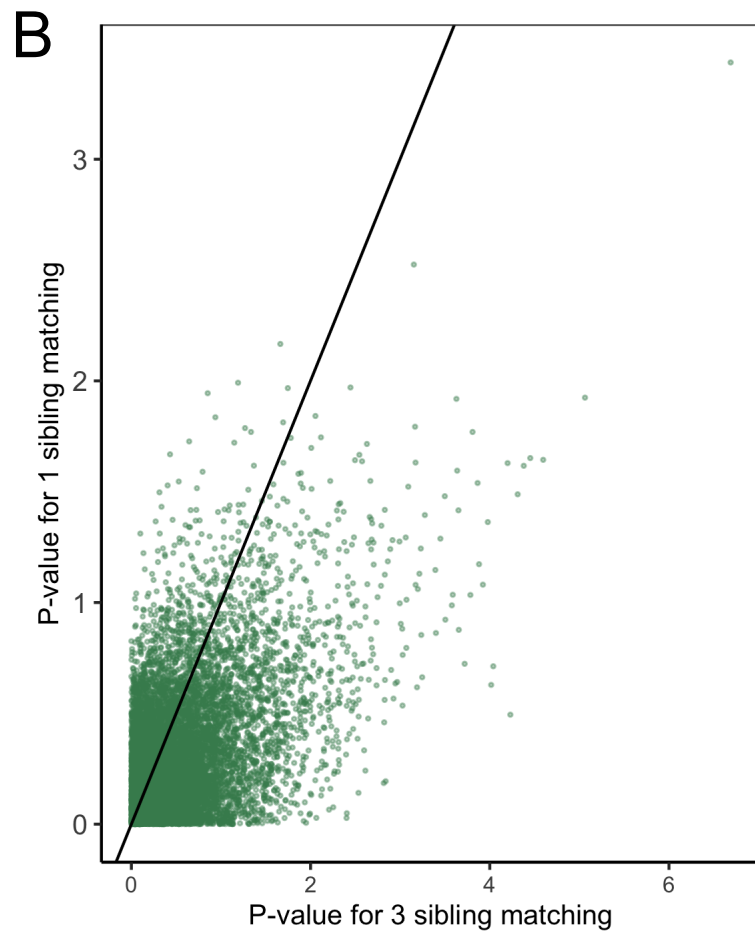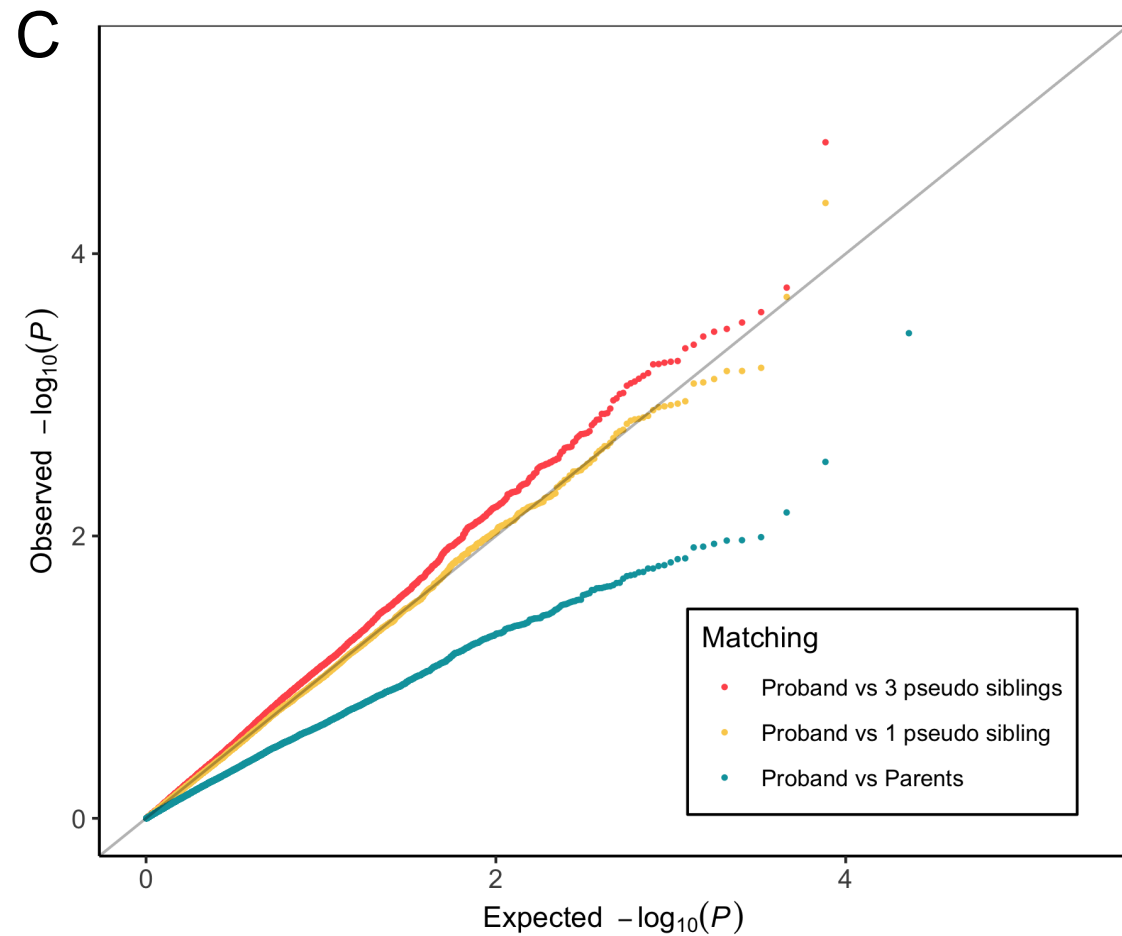

Supplement: S18 Fig — (A) The −log10 P values between 3-sibling and 1-sibling matching. (B) The −log10 P values between 3-sibling and parent-control matching. (C) The QQ plots for 3-sibling, 1-sibling, and parent-control matching. (PDF) [file pgen.1009309.s018.pdf]

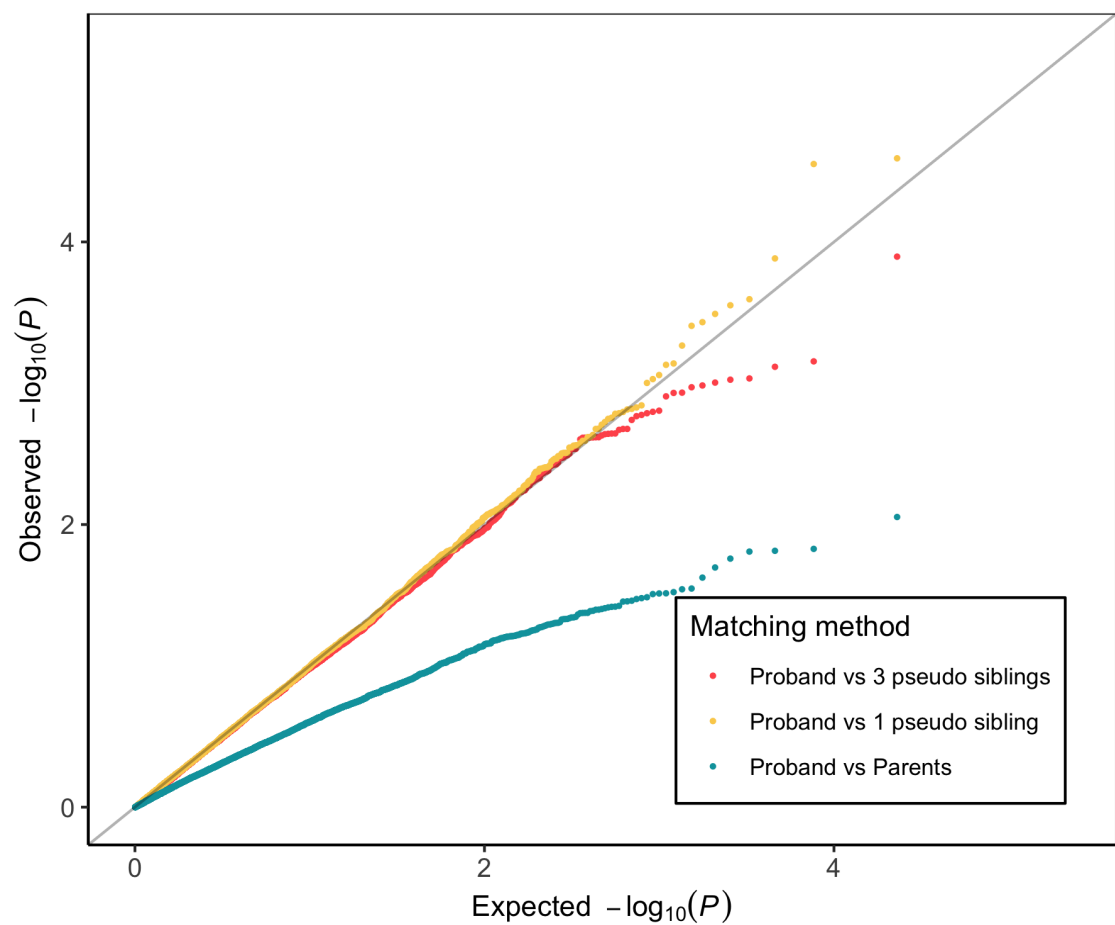

Supplement: S19 Fig — The QQ plot for 3-sibling, 1-sibling, and parent-control matching performed on 7,805 trios in GTEx hippocampus with shuffled disease status. The 1-sibling matching TWAS is conducted on proband-pseudo sibling pairs where the pseudo siblings were constructed using untransmitted parental alleles. The parent-control matching TWAS is conducted on parents versus a random sample from the quad in 3-sibling matching (Material and Methods). The association results were obtained using conditional logistic regression. (PDF) [file pgen.1009309.s019.pdf]

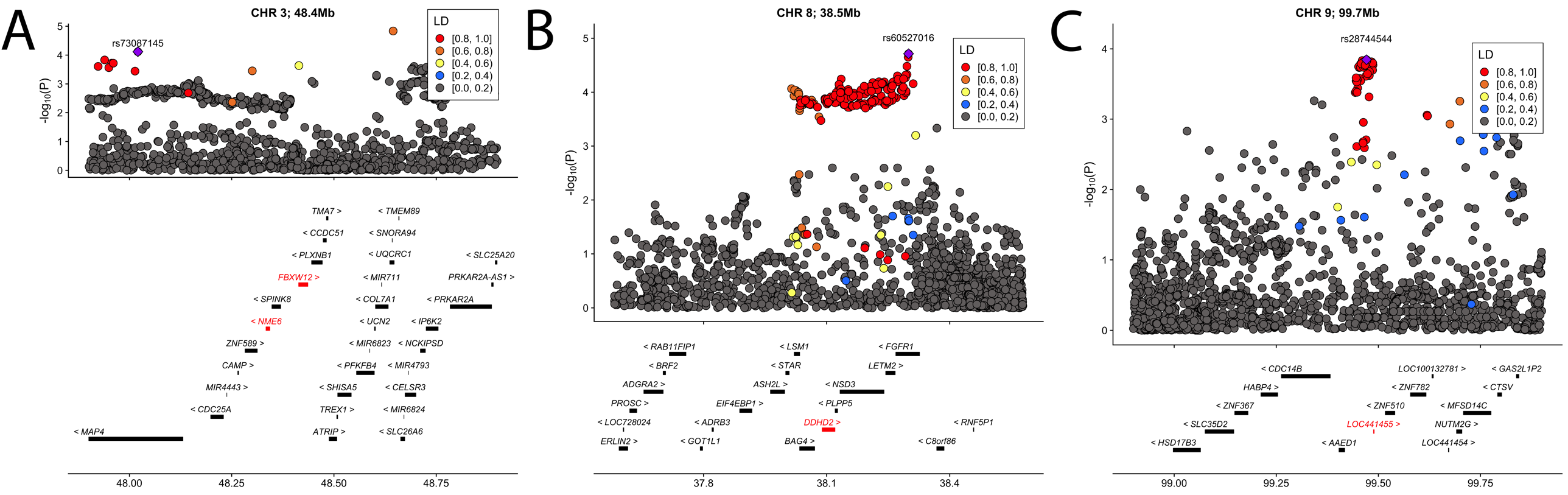

Supplement: S20 Fig — We identified 31 transcriptome-wide significant associations from 7 independent loci. Four loci with associations that remained significant after correcting for all genes and all tissues are shown in Fig 3 in the main text. (A) Chromosome 3, 48.4 mb (B) Chromosome 8, 38.5 mb (C) Chromosome 9, 99.7 mb. For each locus, the index SNP with the most significant association in GWAS is marked as purple diamond and the color of data points indicates LD of neighboring SNPs with the index SNP. Genes are highlighted in red if they reached transcriptome-wide significance in at least one tissue. The x-axis denotes genome coordinates and the y-axis denotes association p-values in GWAS. (PNG) [file pgen.1009309.s020.png]

# Gene expression for *POU3F2*

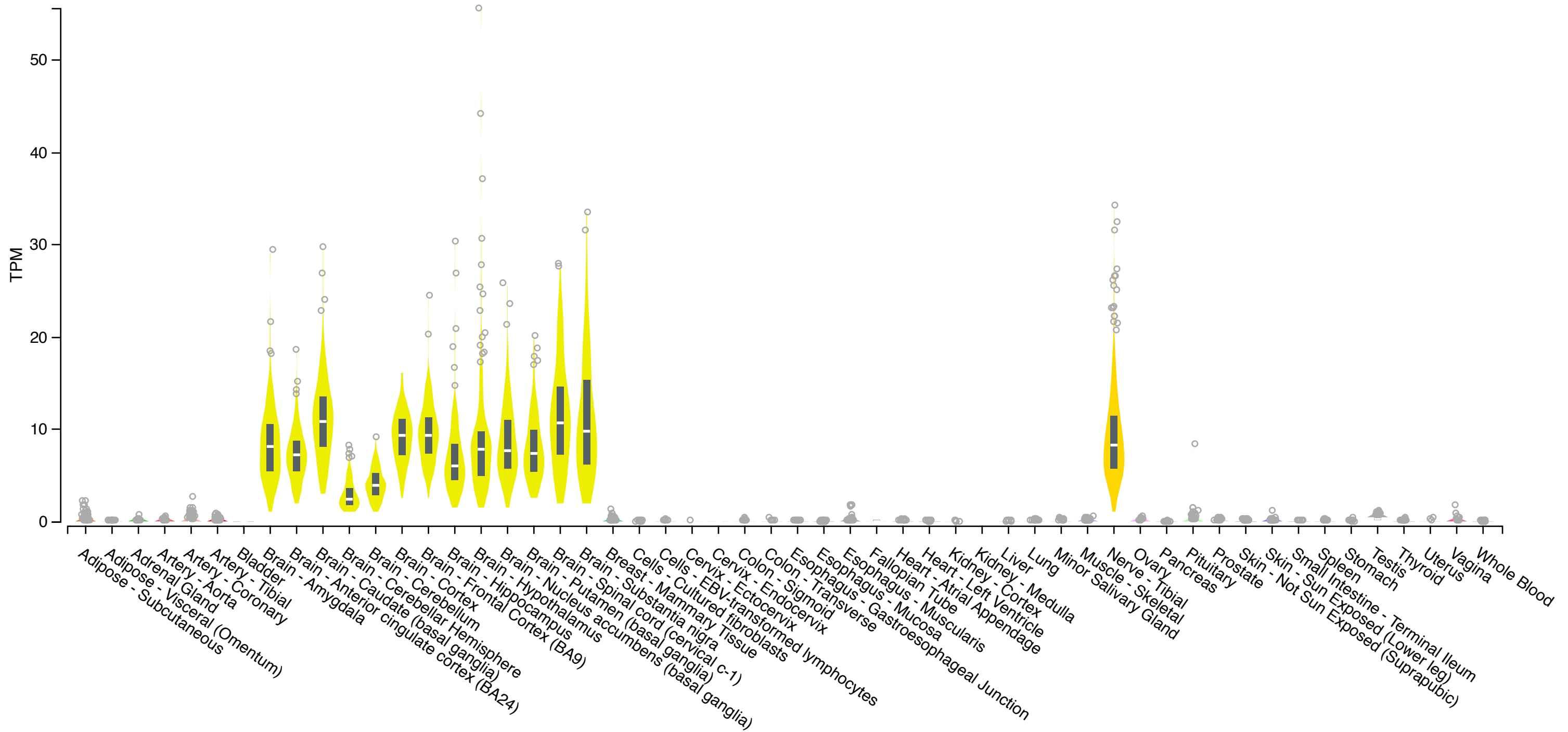

Supplement: S21 Fig — (PDF) [file pgen.1009309.s021.pdf]

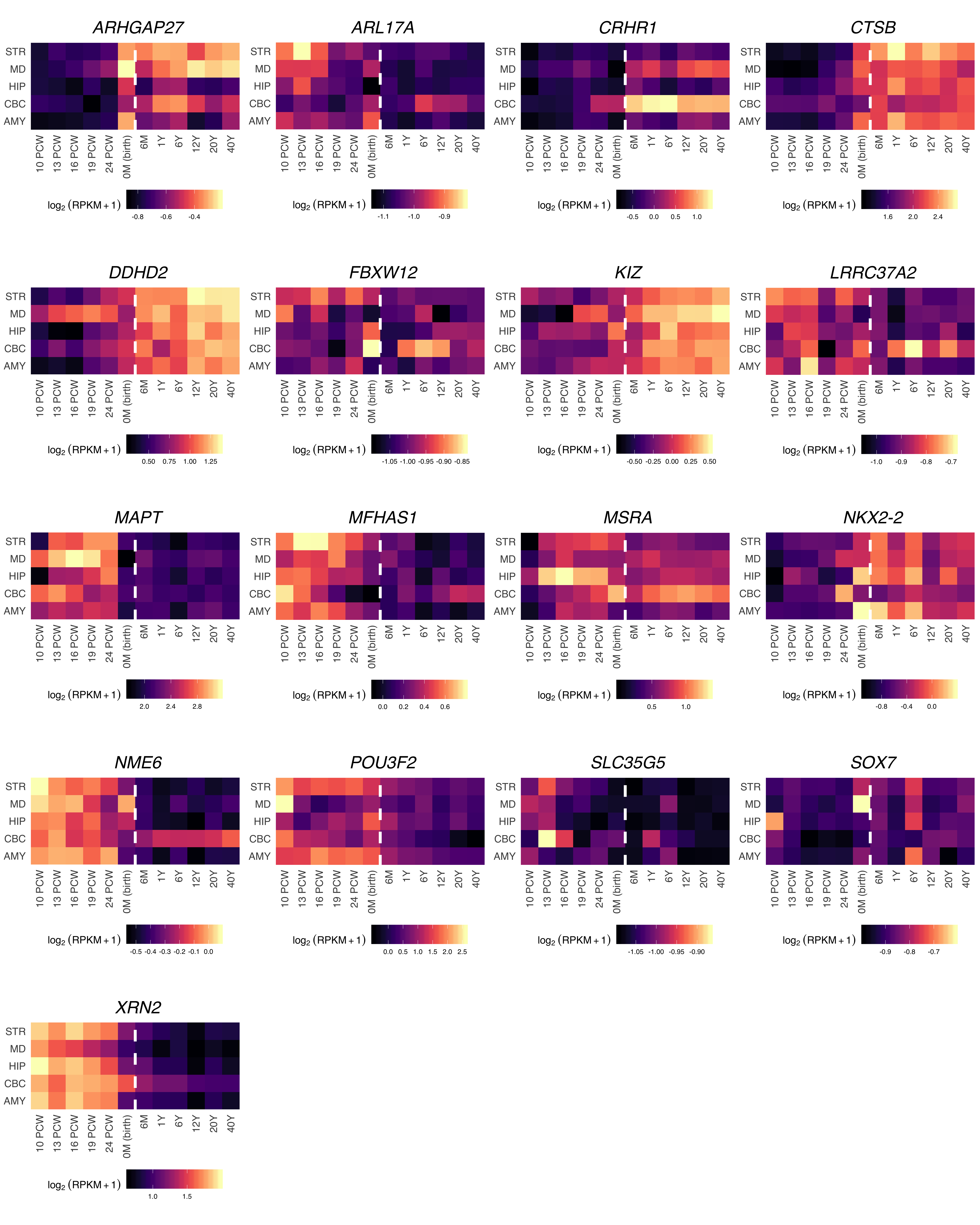

Supplement: S22 Fig — The spatiotemporal expression pattern of 17 TWAS genes across 5 brain regions and 12 developmental stages. The periods span fetal development, infancy, childhood, adolescence, and adulthood, from 4 post-conceptional weeks (PCW) to 40 postnatal years (Y). The dashed line indicates the boundary between later fetal and early infancy stages (0 month). (PNG) [file pgen.1009309.s022.png]

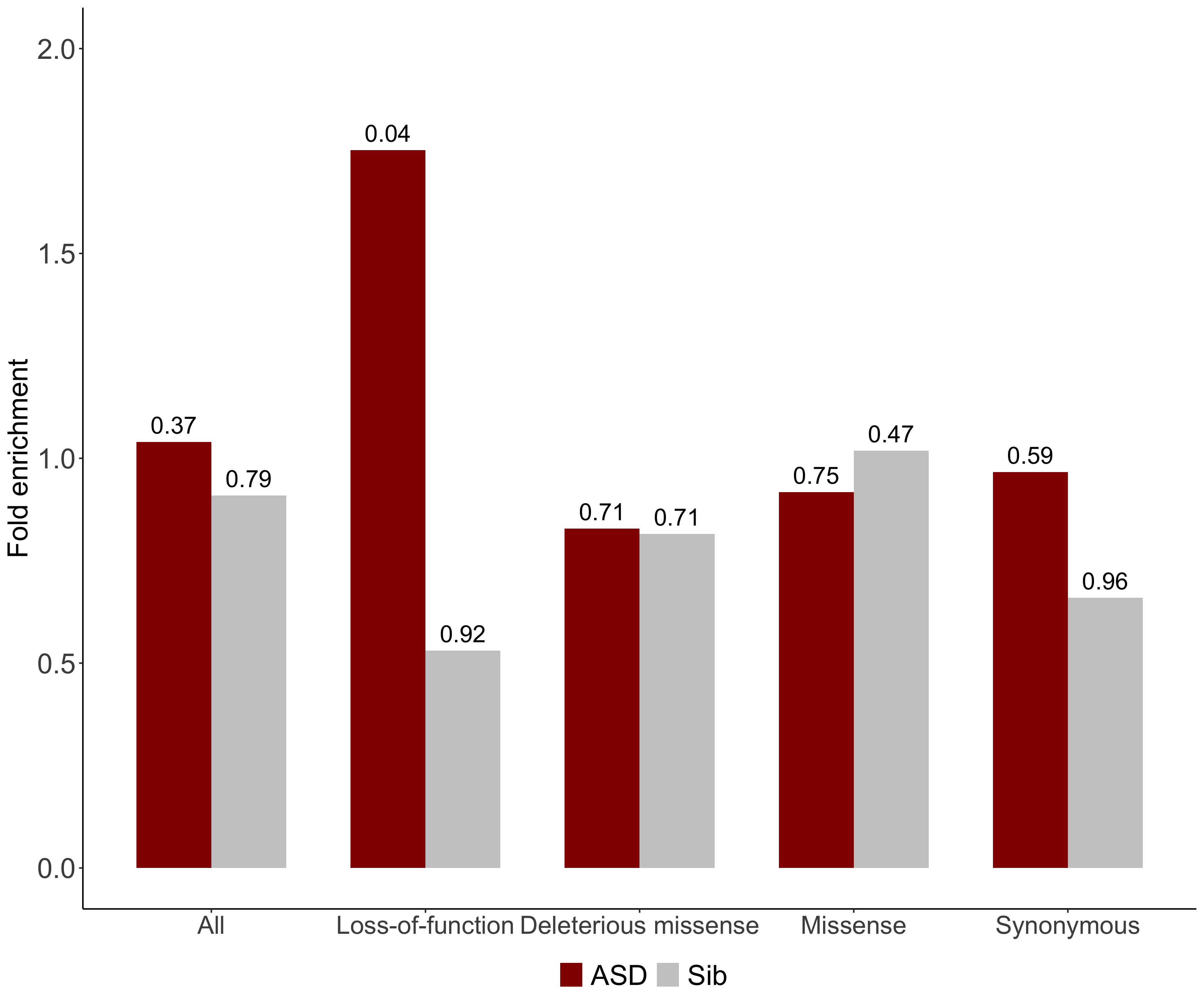

Supplement: S23 Fig — Enrichment results in 2,508 ASD probands and 1,911 unaffected siblings across four annotation categories (all mutations, loss-of-function, missense, deleterious missense, and synonymous) are shown. p-values are shown above each bar. (PNG) [file pgen.1009309.s023.png]
