# Supplementary material for: Transcriptome-wide transmission disequilibrium analysis identifies novel risk genes for autism spectrum disorder
Source: PLoS Genet. 2021 Feb 4;17(2):e1009309. doi: 10.1371/journal.pgen.1009309 (PMC7888619; doi:10.1371/journal.pgen.1009309)
Supplement: S11 Fig — SOX7, MFHAS1, MSRA, CRHR1, MAPT, and XRN2 reached transcriptome-wide significance in the TWAS in CMC DLPFC splicing. Intron cluster IDs are shown below the gene names. Standardized effect sizes (beta) and SEs are provided for the trio-based cohorts. Beta and SE labeled as the discovery cohort are meta-analyzed results based on AGP, SSC, and SPARK. Effect estimates are not shown in the replication and the combined cohorts since FUSION does not output beta and SE estimates. (PDF) [file pgen.1009309.s011.pdf]

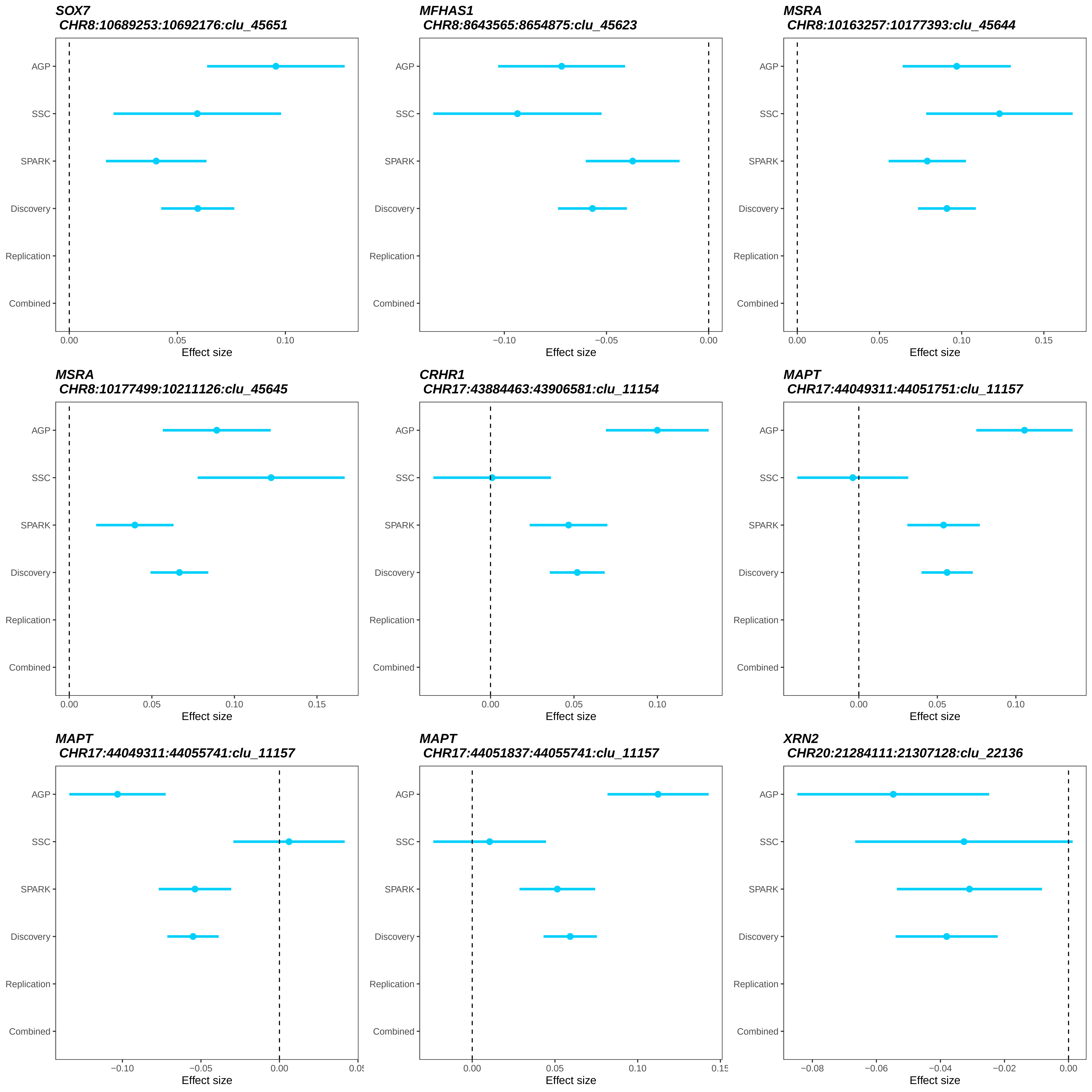
SOX7
CHR8:10689253:10692176:clu_45651
MFHAS1
CHR8:8643565:8654875:clu_45623
MSRA
CHR8:10163257:10177393:clu_45644
MSRA
CHR8:10177499:10211126:clu_45645
CRHR1
CHR17:43884463:43906581:clu_11154
MAPT
CHR17:44049311:44051751:clu_11157
MAPT
CHR17:44049311:44055741:clu_11157
MAPT
CHR17:44051837:44055741:clu_11157
XRN2
CHR20:21284111:21307128:clu_22136
AGP
SSC
SPARK
Discovery
Replication
Combined
Effect size
